# Supplementary material for: LptM promotes oxidative maturation of the lipopolysaccharide translocon by substrate binding mimicry
Source: Nat Commun. 2023 Oct 11;14:6368. doi: 10.1038/s41467-023-42007-w (PMC10567701; doi:10.1038/s41467-023-42007-w)
Supplement: Supplementary file 1 — Supplementary Information [file 41467_2023_42007_MOESM1_ESM.pdf]

**SUPPLEMENTARY INFORMATION**

**LptM promotes oxidative maturation of the lipopolysaccharide translocon by substrate binding mimicry.**

Yiying Yang, Haoxiang Chen, Robin A. Corey, Violette Morales, Yves Quentin, Carine Froment, Anne Caumont-Sarcos, Cécile Albenne, Odile Burlet-Schiltz, David Ranava, Phillip J. Stansfeld, Julien Marcoux, Raffaele Ieva

**Supplementary Information includes:**

Supplementary Methods

Supplementary Figures 1-15

Supplementary Tables 1-3

Supplementary References

## Supplementary Methods

### Genome samples for taxonomic analysis.

A representative set of 2927 bacterial genomes was assembled by selecting one genome per bacterial Family from the Genome Taxonomy Database (GTDB) [1] and downloaded from the NCBI website (ftp://ftp.ncbi.nlm.nih.gov/genomes). In GTDB, Beta-proteobacteria are classified as an order within the class Gamma-proteobacteria. Representative set of Alpha- and Gamma-proteobacteria down to the Genera level and *Enterobacteriaceae* samples down to the Genera and Species level were also built. All genome accession codes and metadata are available in the Source Data file.

### Search of LptM-like candidates.

Genomes were annotated with *Prokka* in fast mode with default settings [2]. Annotation of proteins with Pfam domains (version 34.0) was performed with *hmmscan* (*HMMER* package version 3.1b2, [3]) and results were filtered to keep only the best non-overlapping alignments. A first analysis showed that genes coding for LptM-like proteins were not always annotated by *Prokka*. Identified candidate sequences were biased in their amino acid composition: the N-terminal regions were enriched in hydrophobic amino acids and the C-terminal regions were reach in amino acid typical of intrinsically disordered protein segments.

Genes located at the ends of contigs may have partial sequences. To obviate these problems, we translated the genomes in all six phases with an ORF size greater than or equal to 10 nucleotides to maximize the probability of identifying candidate genes (*esl-translate* program from *HMMER* package). ORFs internal to *Prokka* annotated genes were excluded from the analysis.

Initially, the presence of the PF13627 domain was used to identify LptM protein candidates in the translated ORFs (trORFs) obtained using *hmmsearch* (from *HMMER* package -E 1). The results indicated that *lptM* gene candidates are generally present in a single copy per genome. By selecting only the sequences with the lowest E-value (one per genome), the sample size was reduced to 985

hits, of which 292 are present in the 476 proteobacterial genomes and 693 in the 2451 non-proteobacterial genomes.

In a second step, *hmmscan* (from *HMMER* package) was used with the Pfam library (version 34.0) against the 985 trORFs selected with the PF13627 profile. 911 trORFs have PF13627 as the best profile which eliminates 74 sequences. The latter sequences are more frequent in non-proteobacterial genomes (9.8% *vs.* 2%) and are mainly annotated as lipoproteins. We estimated the length of the C-terminal region (defined as the amino acid sequence of protein downstream of the PF13627 domain) by subtracting the final position of the alignment with the PF13627 domain from the sequence size. The vast majority of candidate proteins annotated in Alpha- and Gamma-proteobacteria have short C-terminal region (<70 amino acids), whereas non-proteobacterial candidates present a longer C-terminal region (Supplementary Fig. 3a). We filtered out candidates with a C-terminal region length greater than or equal to 70 amino acids. In Supplementary Fig. 3b are plotted the distribution of scores and alignment lengths for the three groups of genomes.

Genomic context of proteobacterial *lptM* gene candidates was extracted from GFF3 *Prokka* annotation files over a 5000 nucleotides window upstream and downstream of these genes. A classification of the proteins encoded by the neighboring genes into groups of homologous sequence was performed with *mmseqs2* [4], followed by a partition of the graph in communities with the Leiden method [5] of the *igraph* package (https://igraph.org/r/). The genes of two most frequent protein clusters are located downstream and upstream the *lptM*-like genes. The average gap between gene clusters and candidate genes is less than 32 nucleotides, suggesting that in the majority of genomes these genes are part of the same operon. The first cluster belongs to the Orn/Lys/Arg decarboxylase class-II family as suggested by the presence of the domain PF02784. The second cluster include genes encoding Lyases (PF00206). This conservation of neighboring gene is more common in Gamma- than Alpha-proteobacteria (Supplementary Fig. 3c). A phylogenetic tree was calculated on the 2927 genomes of our bacterial sample (Supplementary Fig. 3d). The alignment of the 120 markers of these

genomes was retrieved from the GTDB. The tree was inferred with *fasttree* [6]. Branch supports were estimated with the local bootstrapping. The logoplots (Supplementary Fig. 3e) of the LptM-like proteins identified in Gamma-, Alpha-, and non-proteobacteria were calculated with *R* package *ggseqlogo* [7]. LptM-like proteins from proteobacteria and non-proteobacteria are reported in Supplementary Data 4 and 5, respectively.

To obtain a more accurate picture of the distribution and evolution of *lptM* in *Enterobacteriaceae*, we extended our analysis to 766 *Enterobacteriaceae* species. We identified 545 LptM-like proteins. To characterize the C-terminal regions of LptM, we used the *meme* software (-protein -mod zoops -nmotifs 25 -minw 4 -maxw 16 -minsites 3 -evt 0.05), which identifies blocks of conserved motifs in a subset of sequences [8]. To obtain a better taxonomic representation, we selected one sequence from each genus (93 sequences). The motifs detected by *meme* were annotated on the whole sequences with the *mast* software [8]. The maximum motif size of 16 AA covers the lipobox and the downstream conserved region (Supplementary Fig. 4).

**Analysis of LptM conservation in *Enterobacteriaceae.***

In Alpha- and Gamma-proteobacteria, two genes are frequently conserved upstream and downstream of the *lptM*-like genes (Supplementary Fig. 3c). This conservation of a chromosomal neighboring genes surrounding *lptM* candidates suggests that they were inherited from a common ancestor and are therefore orthologs. This reinforces the hypothesis that, in proteobacteria, the PF13627 domain reliably identifies proteins that have a common origin and similar functions. This also suggests that the proteins predicted from the translated ORFs are functional.

The distribution of *lptM*-like genes in bacteria shows that they are very frequent and with high scores in Gamma-proteobacteria (in 82.1% of genomes), less frequent with lower scores in Alpha-proteobacteria (61.4%) and infrequent (9.1%) with very low scores in non-proteobacteria (Supplementary Fig. 3d). By filtering the results with a thresholds score ≥ 13 and an alignment length score ≥ 16, the

number of candidates in non-proteobacteria and in Alpha-proteobacteria is significantly reduced whereas the number of candidates in Gamma-proteobacteria is marginally reduced (1.7%, 56.6% and 78.9%, respectively). In agreement with these observations, LptM candidates from Gamma-proteobacteria show more extensive sequence conservation downstream of the lipobox cysteine (Supplementary Fig. 3e). In addition, *lptM*-like genes appear to be randomly distributed in non-proteobacterial genomes. This distribution and the low sequence scores suggest that they do not encode for a true homolog of proteobacterial LptM. However, these non-proteobacterial proteins have lipoprotein characteristics captured by the PF13627 profile.

We identified the LptD, LptE, and LptM proteins in the genomes of 766 species of *Enterobacteriaceae*. The results are summarized in Supplementary Fig. 4, where only the 135 representative genomes of the GTDB *Enterobacteriaceae* genera are shown. Based on phylogenetic analyses and the use of conserved molecular features it was proposed to divide the *Enterobacteriaceae* family into seven new families [9]: a restricted *Enterobacteriaceae* family, and the new families Erwiniaceae, Pectobacteriaceae, Yersiniaceae, Hafniaceae, Morganellaceae and Budviciaceae. The newly defined family of restricted *Enterobacteriaceae* was subdivided into 6 subfamilies: *Escherichia*, *Klebsiella*, *Enterobacter*, *Kosakonia*, *Cronobacter*, *Cedecea*, and an "*Enterobacteriaceae incertae sedis*" clade containing species whose taxonomic placement within the family is not clear [10]. The phylogenetic tree reconstructed on the 135 representative genomes is in agreement with this classification (Supplementary Fig. 4). The *lptM* annotations are available in the Source Data file, and the LptM amino acid sequences are available in Supplementary Data 6.

As above, LptM-coding genes are not always annotated but can be identified as ORFs. We annotated LptD and LptE proteins in these genomes using PF04453 and PF04390 profiles. The three proteins are co-occurring in the vast majority of genomes larger than $2x10^6$ nucleotides, while they are present in only 6 of 33 genomes smaller than $2x10^6$ nucleotides. In these genomes LptM is most often absent and LptD is most often present. The bacteria that underwent a strong re-

duction of their genome size belong to subtrees that include endosymbiotic bacteria (Supplementary Fig. 4). The reduction in genome size is accompanied by the loss of a large number of genes, so the simultaneous loss of the genes encoding the three Lpt proteins in 14 out of 33 genomes provides only weak evidence for the existence of a functional link between these three proteins.

Two *meme* motifs are found exclusively in the LptM proteins of genomes belonging to the newly defined restricted *Enterobacteriaceae* family (Supplementary Fig. 4). This distribution suggests that they were acquired in the last common ancestor of this subfamily, either in a single event or stepwise. The presence of these motifs stabilized the C-terminal region which is generally variable in length and sequence in other LptM proteins. The third motif which is rich in polar amino acids (Q, N, T and S) is present in proteins of other families with a low frequency (18/70).

Outside the *Enterobacteriaceae* family, LptM proteins lack sequence conservation for the C-terminal region, suggesting that either this segment of LptM does not mediate specific protein-protein interactions in these bacteria or that the nature of these interactions is not evolutionarily conserved. Instead, the sequences of LptM in the restricted *Enterobacteriaceae* family presents several distinguishing motifs including the presence of a conserved C-terminal region, suggesting a relatively recent acquisition.

**Three-dimensional representation of sequence conservation scores.**

Multiple alignments of the three protein families (LptE, LptD and LptM) were obtained from the 37 genomes of the Enterobacteriaceae family shown in Supplementary Fig. 4, to which the *E. coli* K-12 sequence were added. Conservation scores were calculated using Chimera [11]. Multi-align viewer conservation scores were then projected onto the proteins three-dimensional structures for visualisation.

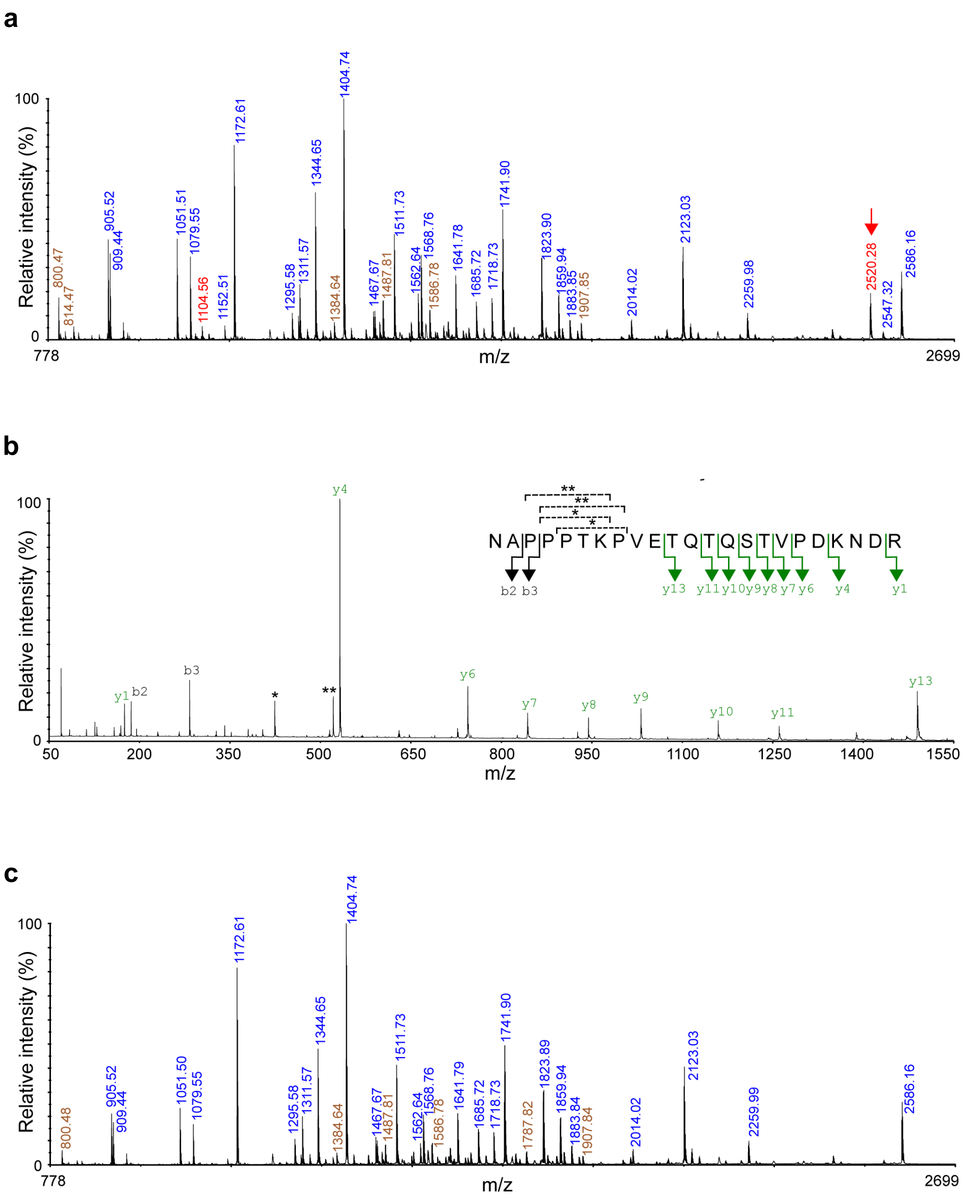


**Supplementary Figure 1. MALDI-TOF mass-spectrometry analysis of purified LptDE$^{His}$.**

**a, c)**. Gel bands obtained by BN-PAGE of the LptDE$^{His}$ complex purified from wild-type (a) or Δ*lptM* (c) cells were subjected to in-gel trypsin digestion and MALDI-TOF analyses. LptD and LptE were identified by peptide mass fingerprinting. The m/z values of peaks matching LptD or LptE peptides are indicated in

blue and brown respectively (a and c). Comparison of the two MS spectra led to the identification of discriminant peaks between the wild-type and the Δ*lptM* samples (m/z values in red). Source data are provided as Source Data file.

**b)** MALDI TOF/TOF fragmentation of ion parent at m/z= 2520.28 (indicated by a red arrow) allowed the identification of the peptide $_{34}$NAPPPTKPVETQTQSTVPDKNDR$_{56}$ of LptM. y and b ions resulting from fragmentation are indicated (central spectrum). Internal fragment ions are labeled with one or two asterisks. Source data are provided as Source Data file.

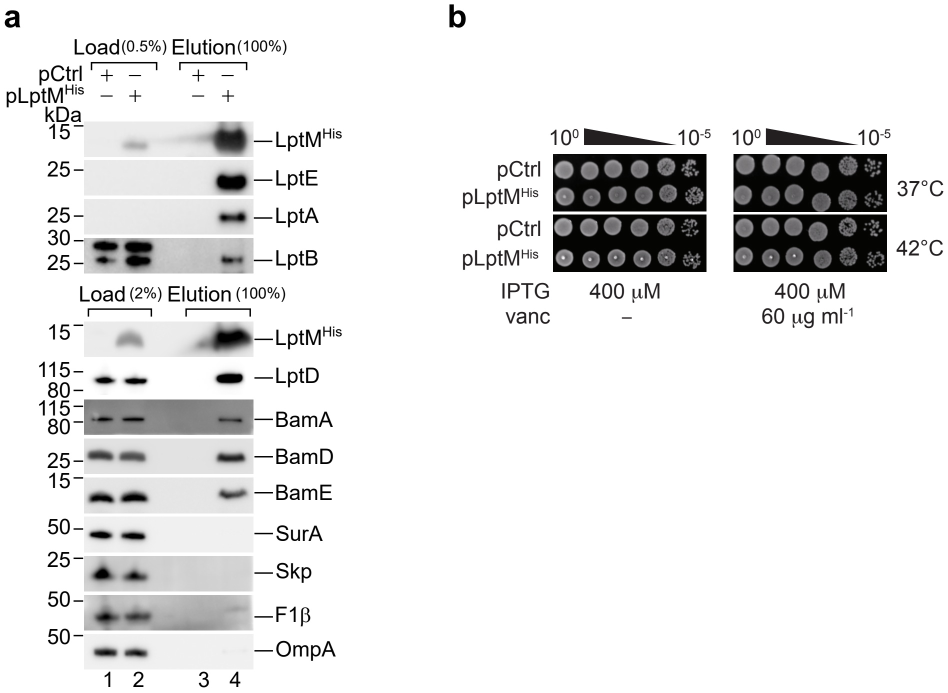


**Supplementary Figure 2. LptM interacts with Lpt and BAM components.**

**a)** DDM-solubilized $LptM^{His}$ was purified from Δ*lptM* cells transformed either with an empty vector pCtrl or $pLptM^{His}$ and analyzed by SDS-PAGE and Western blotting using the indicated antisera. Load: 0.5% or 2%, as indicated; Elution: 100%. Note that the levels of LptE and LptA are below the threshold of detection in the load fractions and clearly enriched in the elution fraction of $LptM^{His}$. Data are representative of triplicate experiments.

**b)** Drop dilution growth test of wild-type *E. coli* transformed with the empty vector pCtrl and Δ*lptM* transformed with $pLptM^{His}$ in the presence of the indicated concentrations of IPTG and vancomycin (vanc). Data are representative of three independent experiments.

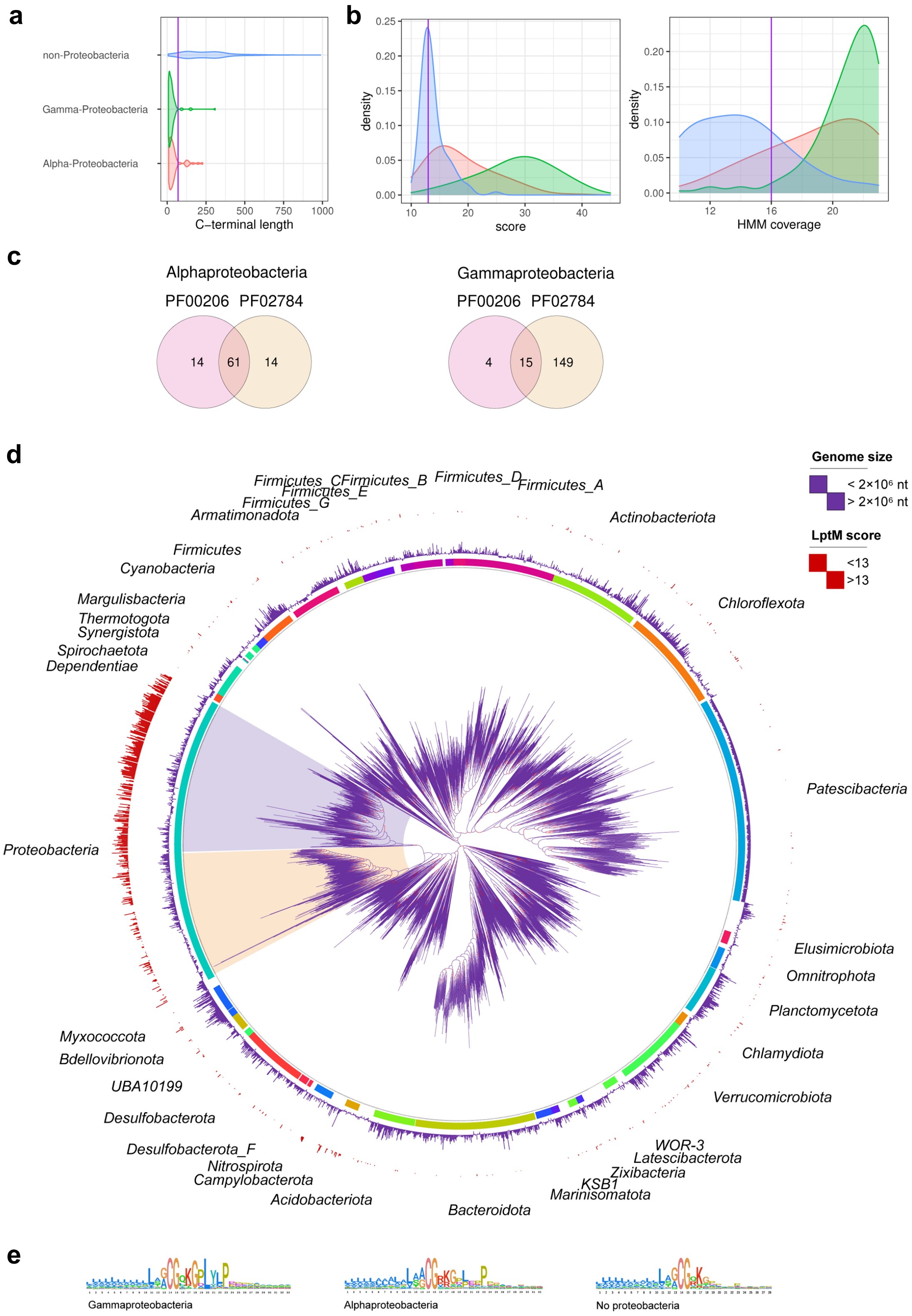

a
non-Proteobacteria
Gamma-Proteobacteria
Alpha-Proteobacteria
C−terminal length
b
density
score
HMM coverage
c
Alphaproteobacteria
PF00206 PF02784
14 61 14
Gammaproteobacteria
PF00206 PF02784
4 15 149
d
Genome size
< 2×10⁶ nt
> 2×10⁶ nt
LptM score
<13
>13
Firmicutes_C Firmicutes_B Firmicutes_D Firmicutes_A
Firmicutes_E
Firmicutes_G
Armatimonadota
Actinobacteriota
Firmicutes
Cyanobacteria
Margulisbacteria
Thermotogota
Synergistota
Spirochaetota
Dependentiae
Chloroflexota
Patescibacteria
Proteobacteria
Elusimicrobiota
Omnitrophota
Planctomycetota
Chlamydiota
Verrucomicrobiota
Myxococcota
Bdellovibrionota
UBA10199
Desulfobacterota
Desulfobacterota_F
Nitrospirota
Campylobacterota
Acidobacteriota
Bacteroidota
WOR-3
Latescibacterota
Zixibacteria
KSB1
Marinisomatota
e
Gammaproteobacteria
Alphaproteobacteria
No proteobacteria

**Supplementary Figure 3. Distribution of LptM-like proteins in bacteria.**

LptM-like proteins were predicted in the translated ORFs of represented families of bacteria with *hmmsearch* and the PF13627 domain.

**a)** Length distribution of the C-terminal region (downstream of PF13627) of the LptM putative amino acid sequences identified in non-proteobacteria, Gamma- and Alpha-proteobacteria (vertical bar at 70 amino acids).

**b)** Alignment score and length distribution in the three samples. Vertical bars indicate strict score (13) and length (16) thresholds.

**c)** Venn diagram with the occurrence of PF00206 and PF02784 protein-coding genes in the neighborhood of *lptM*-like genes in Alpha and Gamma-proteobacteria.

**d)** Inferred phylogenetic tree for representative bacterial families with *fasttree* from the alignment of 120 markers retrieved from GTDB. The color gradient of the branches is proportional to the local bootstrap support values from 0 (red) to 1 (purple). Alpha and Gamma-proteobacteria clades are indicated on the tree (yellow and blue shading of subtrees). To simplify the annotation, only phyla that contain at least 10 genomes have been indicated in the figure on the first ring with a color code and their names are reported in the outer ring. Genome size is shown as a purple histogram with a threshold at $2x10^6$ nucleotides, i.e., genomes smaller than $2x10^6$ nucleotides are shown as negative. The next ring shows the value of the LptM-like protein score with a threshold of 13 (scores below 13 are shown as negative). Source data relative to the bacterial genomes used for the phylogenetic tree are provided as Source Data file.

**e)** Sequence logos of the N-terminal region of LptM-like proteins identified in Gamma-, Alpha- and non-proteobacteria.

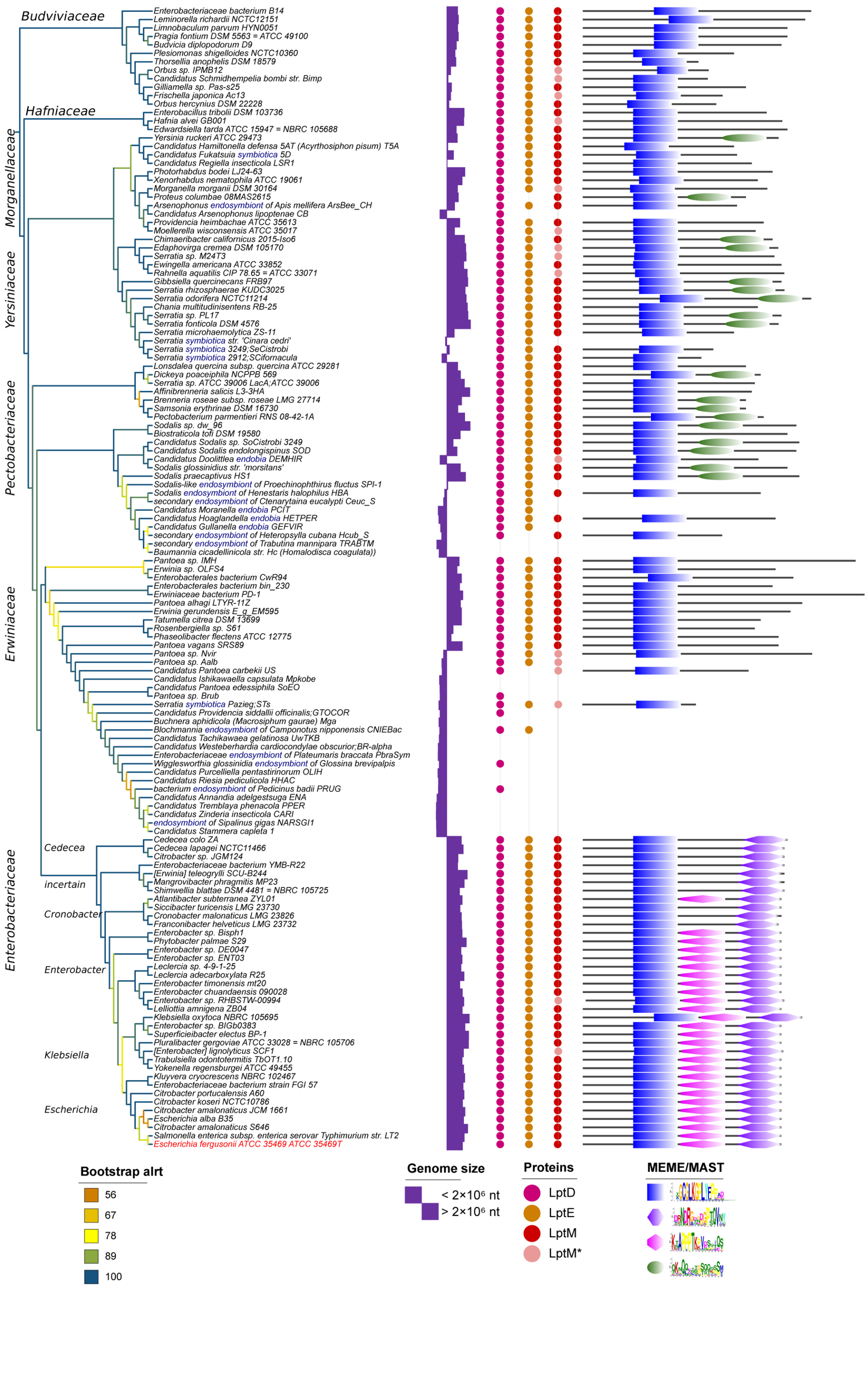

Budviviaceae
Hafniaceae
Morganellaceae
Yersiniaceae
Pectobacteriaceae
Erwiniaceae
Enterobacteriaceae
Cedecea
incertain
Cronobacter
Enterobacter
Klebsiella
Escherichia
Enterobacteriaceae bacterium B14
Leminorella richardii NCTC12151
Limnobaculum parvum HYN0051
Pragia fontium DSM 5563 = ATCC 49100
Budvicia diplopodorum D9
Plesiomonas shigelloides NCTC10360
Thorsellia anophelis DSM 18579
Orbus sp. IPMB12
Candidatus Schmidhempelia bombi str. Bimp
Gilliamella sp. Pas-s25
Frischella japonica Ac13
Orbus hercynius DSM 22228
Enterobacillus tribolii DSM 103736
Hafnia alvei GB001
Edwardsiella tarda ATCC 15947 = NBRC 105688
Yersinia ruckeri ATCC 29473
Candidatus Hamiltonella defensa 5AT (Acyrthosiphon pisum) T5A
Candidatus Fukatsuia symbiotica 5D
Candidatus Regiella insecticola LSR1
Photorhabdus bodei LJ24-63
Xenorhabdus nematophila ATCC 19061
Morganella morganii DSM 30164
Proteus columbae 08MAS2615
Arsenophonus endosymbiont of Apis mellifera ArsBee_CH
Candidatus Arsenophonus lipoptenae CB
Providencia heimbachae ATCC 35613
Moellerella wisconsensis ATCC 35017
Chimaeribacter californicus 2015-Iso6
Edaphovirga cremea DSM 105170
Serratia sp. M24T3
Ewingella americana ATCC 33852
Rahnella aquatilis CIP 78.65 = ATCC 33071
Gibbsiella quercinecans FRB97
Serratia rhizosphaerae KUDC3025
Serratia odorifera NCTC11214
Chania multitudinisentens RB-25
Serratia sp. PL17
Serratia fonticola DSM 4576
Serratia microhaemolytica ZS-11
Serratia symbiotica str. 'Cinara cedri'
Serratia symbiotica 3249;SeCistrobi
Serratia symbiotica 2912;SCifornacula
Lonsdalea quercina subsp. quercina ATCC 29281
Dickeya poaceiphila NCPPB 569
Serratia sp. ATCC 39006 LacA;ATCC 39006
Affinibrenneria salicis L3-3HA
Brenneria roseae subsp. roseae LMG 27714
Samsonia erythrinae DSM 16730
Pectobacterium parmentieri RNS 08-42-1A
Sodalis sp. dw_96
Biostraticola tofi DSM 19580
Candidatus Sodalis sp. SoCistrobi 3249
Candidatus Sodalis endolongispinus SOD
Candidatus Doolittlea endobia DEMHIR
Sodalis glossinidius str. 'morsitans'
Sodalis praecaptivus HS1
Sodalis-like endosymbiont of Proechinophthirus fluctus SPI-1
Sodalis endosymbiont of Henestaris halophilus HBA
secondary endosymbiont of Ctenarytaina eucalypti Ceuc_S
Candidatus Moranella endobia PCIT
Candidatus Hoaglandella endobia HETPER
Candidatus Gullanella endobia GEFVIR
secondary endosymbiont of Heteropsylla cubana Hcub_S
secondary endosymbiont of Trabutina mannipara TRABTM
Baumannia cicadellinicola str. Hc (Homalodisca coagulata))
Pantoea sp. IMH
Erwinia sp. OLFS4
Enterobacterales bacterium CwR94
Enterobacterales bacterium bin_230
Erwiniaceae bacterium PD-1
Pantoea alhagi LTYR-11Z
Erwinia gerundensis E_g_EM595
Tatumella citrea DSM 13699
Rosenbergiella sp. S61
Phaseolibacter flectens ATCC 12775
Pantoea vagans SRS89
Pantoea sp. Nvir
Pantoea sp. Aalb
Candidatus Pantoea carbekii US
Candidatus Ishikawaella capsulata Mpkobe
Candidatus Pantoea edessiphila SoEO
Pantoea sp. Brub
Serratia symbiotica Pazieg;STs
Candidatus Providencia siddallii officinalis;GTOCOR
Buchnera aphidicola (Macrosiphum gaurae) Mga
Blochmannia endosymbiont of Camponotus nipponensis CNIEBac
Candidatus Tachikawaea gelatinosa UwTKB
Candidatus Westeberhardia cardiocondylae obscurior;BR-alpha
Enterobacteriaceae endosymbiont of Plateumaris braccata PbraSym
Wigglesworthia glossinidia endosymbiont of Glossina brevipalpis
Candidatus Purcelliella pentastirinorum OLIH
Candidatus Riesia pediculicola HHAC
bacterium endosymbiont of Pedicinus badii PRUG
Candidatus Annandia adelgestsuga ENA
Candidatus Tremblaya phenacola PPER
Candidatus Zinderia insecticola CARI
endosymbiont of Sipalinus gigas NARSGI1
Candidatus Stammera capleta 1
Cedecea colo ZA
Cedecea lapagei NCTC11466
Citrobacter sp. JGM124
Enterobacteriaceae bacterium YMB-R22
[Erwinia] teleogrylli SCU-B244
Mangrovibacter phragmitis MP23
Shimwellia blattae DSM 4481 = NBRC 105725
Atlantibacter subterranea ZYL01
Siccibacter turicensis LMG 23730
Cronobacter malonaticus LMG 23826
Franconibacter helveticus LMG 23732
Enterobacter sp. Bisph1
Phytobacter palmae S29
Enterobacter sp. DE0047
Enterobacter sp. ENT03
Leclercia sp. 4-9-1-25
Leclercia adecarboxylata R25
Enterobacter timonensis mt20
Enterobacter chuandaensis 090028
Enterobacter sp. RHBSTW-00994
Lelliottia amnigena ZB04
Klebsiella oxytoca NBRC 105695
Enterobacter sp. BIGb0383
Superficieibacter electus BP-1
Pluralibacter gergoviae ATCC 33028 = NBRC 105706
[Enterobacter] lignolyticus SCF1
Trabulsiella odontotermitis TbOT1.10
Yokenella regensburgei ATCC 49455
Kluyvera cryocrescens NBRC 102467
Enterobacteriaceae bacterium strain FGI 57
Citrobacter portucalensis A60
Citrobacter koseri NCTC10786
Citrobacter amalonaticus JCM 1661
Escherichia alba B35
Citrobacter amalonaticus S646
Salmonella enterica subsp. enterica serovar Typhimurium str. LT2
Escherichia fergusonii ATCC 35469 ATCC 35469T
Bootstrap alrt
56
67
78
89
100
Genome size
< 2×10⁶ nt
> 2×10⁶ nt
Proteins
LptD
LptE
LptM
LptM*
MEME/MAST

**Supplementary Figure 4. Distribution of LptD, LptE and LptM proteins in *Enterobacteriaceae* and conserved motifs in LptM.**

We used a single representative genome per genus of *Enterobacteriaceae*. Left column, the tree calculated on the alignment of the 120 markers retrieved from GTDB with IQ-TREE (Minh et al. 2020). The trees are plotted with iTOL. The lengths of the branches are not proportional to the distances inferred by IQ-TREE, because genomes (including endosymbionts) with very high evolutionary rates mask the other parts of the tree. The branch supports were assessed with the SH-like approximate likelihood ratio test (-alrt 1000). It was reported on the tree as branch color gradient. The tree is rooted using the tree obtained with the representative genomes of the Bacteria families (Supplementary Fig. 3d). The division of the GTDB family *Enterobacteriaceae* into seven families [9] and the subdivision into six subfamilies of the newly defined family of restricted *Enterobacteriaceae* [10] were reported on the tree. Second column, histogram of the genome sizes. A threshold of $2x10^6$ nucleotides was used to highlight the presence of small genomes. Third column, presence/absence of LptD, LptE and LptM proteins. In the LptM column the dark red circles indicate the presence of an annotated gene and the lighter circles an ORF whose translation product is similar to LptM (LptM*). Last column, LptM mast annotation of the four identified meme domains. The sequence logos of the motifs are indicated. The *Escherichia* genus, represented by *Escherichia fergusoniiis*, is located at the bottom of the tree (colored in red). The terms endosymbiont, endobia and symbiotica present in the genome descriptions are colored in blue. Source data relative to the *Enterobacteriaceae* genomes are provided as Source Data file.

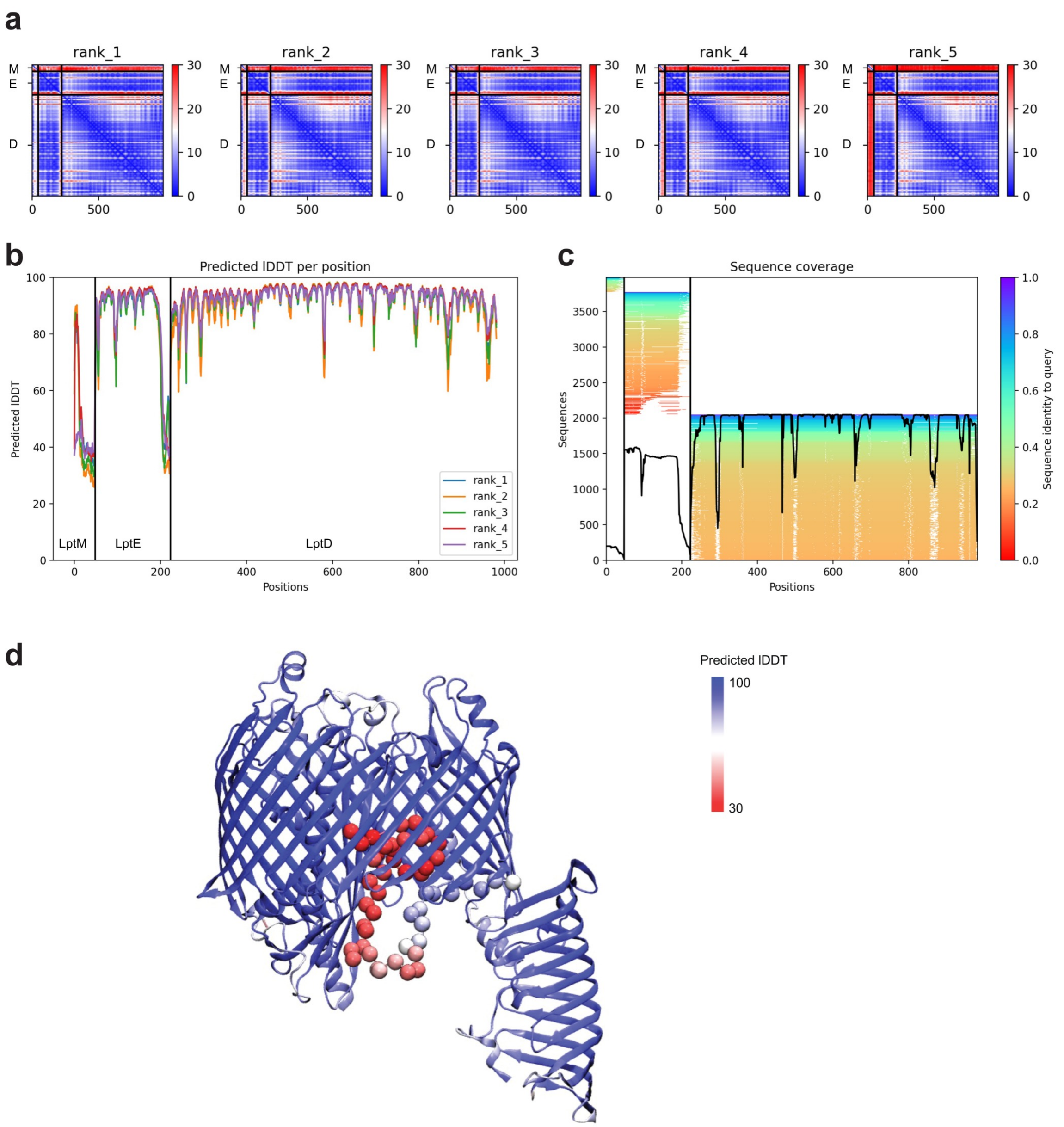
a
rank_1
rank_2
rank_3
rank_4
rank_5
M
E
D
b
Predicted lDDT per position
Predicted lDDT
Positions
LptM
LptE
LptD
c
Sequence coverage
Sequences
Positions
Sequence identity to query
d
Predicted lDDT
100
30

**Supplementary Figure 5. AlphaFold2 output files as produced by Colab-Fold.**

**a)** Predicted Aligned Error (PAE) plots, with the LptM, E, and D subunits marked. Both axes indicate individual residue positions in the three proteins. **b and c)** Positional predicted lDDT scores of the AlphaFold2 models (b) and coverage of the input sequence alignment (c). We refer readers to the AlphaFold2 website (https://alphafold.ebi.ac.uk/) for more information on these analyses. **d)** lDDT scores mapped on the AlphaFold2 structural model of LptDEM. LptM is shown as spheres.

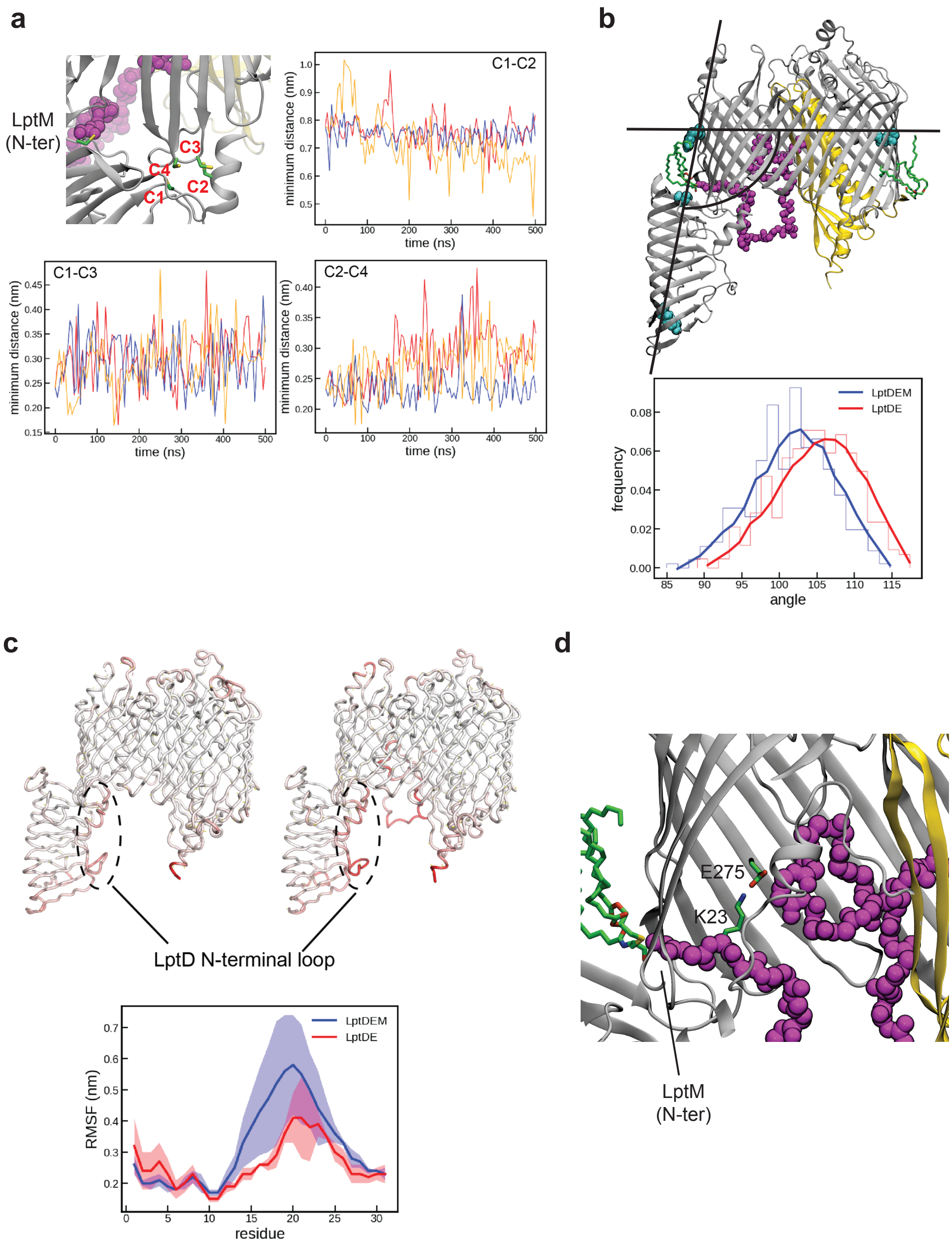


**Supplementary Figure 6. Structure prediction and molecular dynamics simulations of LptDE and LptDEM.**

**a)** A view of the LptD hinge in the LptDE-LptM AphaFold2 model. The N-terminal Cys position of LptM (purple) is shown, as are the predicted positions of LptD C1

to C4. Graphs plot the minimum distance between selected Cys pairs throughout 3 x 500 ns simulations of the LptDEM heterotrimer (each run plotted separately in red, blue or orange).

**b)** Angle of LptD $\beta$-taco in relation to the LptD $\beta$-barrel throughout 3 x 500 ns, as computed using vectors between the residues shown in cyan. The presence of LptM shifts the angles to be lower (more upright).

**c)** Top: C-alpha RMSFs over 3 x 500 ns simulation projected onto the LptDE or LptDE-LptM model. Bottom: RMSF plots focused on the LptD N-terminus, which is destabilized by LptM.

**d)** A zoom-in of the LptDEM heterotrimer modeled using AlphaFold2 (as in Fig. 5a) highlights the interaction of a LptM N-terminal segment with the LptD $\beta$-barrel domain. A salt-bridge is predicted to form between LptM K23 and LptD E275.

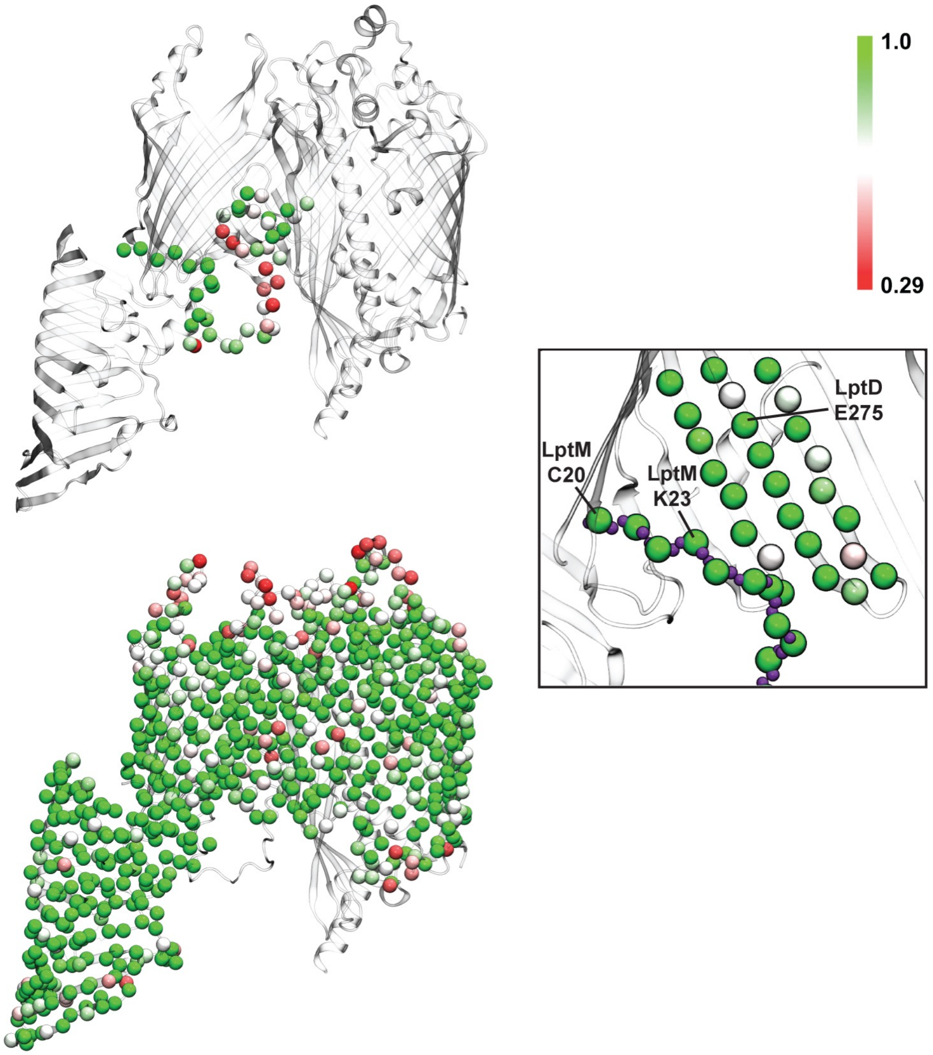


**Supplementary Figure 7. Conservation analyses of LptDEM.**

Cα atoms of LptM (top) and LptD (bottom) AlphaFold2 structures are shown as spheres and coloured by conservation score (see methods). The proteins not coloured by sequence conservation score are shown as white cartoon. The insert shows the LptM N-terminal region and the close LptD β-strands, highlighting the predicted salt bridge between LptM K23 and LptD E275. The LptM backbone is shown as blue spheres and LptD as transparent white cartoon.

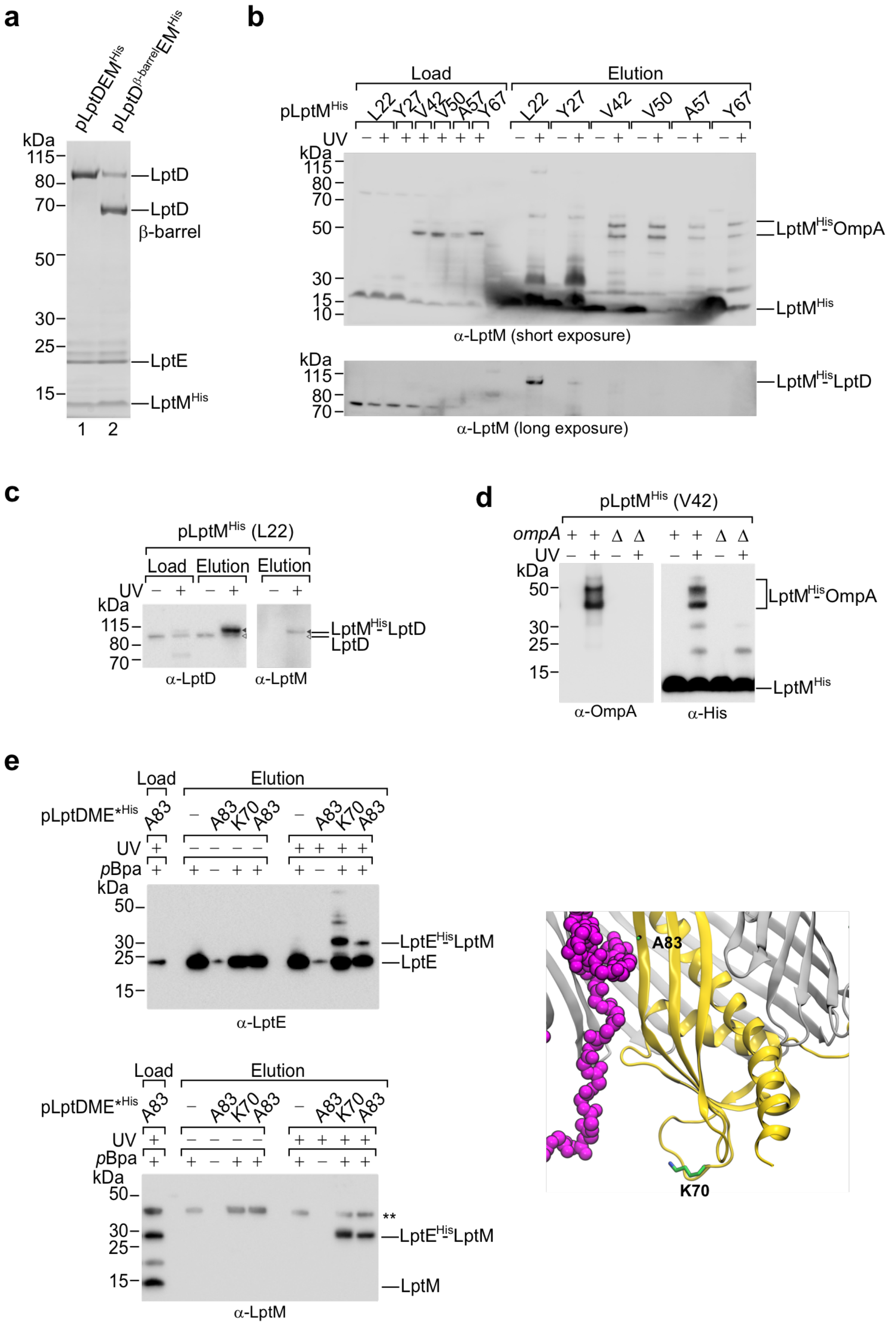

a
pLptDEM^His
pLptD^β-barrel EM^His
kDa
115
80
70
50
30
25
15
LptD
LptD β-barrel
LptE
LptM^His
1 2
b
Load
Elution
pLptM^His
L22 Y27 V42 V50 A57 Y67
UV
α-LptM (short exposure)
LptM^His-OmpA
LptM^His
α-LptM (long exposure)
LptM^His-LptD
c
pLptM^His (L22)
Load Elution Elution
α-LptD
α-LptM
LptM^His-LptD
LptD
d
pLptM^His (V42)
ompA
α-OmpA
α-His
LptM^His-OmpA
LptM^His
e
Load
Elution
pLptDME*^His
A83 K70
pBpa
α-LptE
LptE^His-LptM
LptE
α-LptM
**
LptM
A83
K70

**Supplementary Figure 8**. **LptM interacts with LptE and LptD β-barrel domain.**

**a)** The LPS translocon was purified by Ni-affinity chromatography of $LptM^{His}$ from Δ*lptM* cells transformed with $pLptDEM^{His}$ or $pLptD^{\beta\text{-barrel}}EM^{His}$, as indicated. Data are representative of three independent experiments.

**b)** Δ*lptM* cells harboring pEVOL-pBpF and expressing $LptM^{His}$ derivative forms with *p*Bpa engineered at specific amino acid positions were subjected to UV irradiation as indicated and envelope fractionation (Load) followed by $LptM^{His}$ affinity purification (Elution). Samples were analyzed by SDS-PAGE and Western blotting using the indicated antisera. Load: 2.2%; Elution: 100%. Data are representative of three independent experiments.

**c)** Upon UV irradiation, the envelope (Load) and the elution fractions (Elution) obtained from Δ*lptM* cells expressing $LptM^{His}$ containing *p*Bpa at position L22 were analyzed by SDS-PAGE and Western blotting using anti-LptM and anti-LptD antisera. Load: 2.2%; Elution: 100%. Data are representative of three independent experiments.

**d)** Upon UV irradiation, as indicated, $LptM^{His}$ containing *p*Bpa at position V42 was purified from Δ*lptM* or Δ*lptM ompA::kan* cells and analyzed by SDS-PAGE and Western blotting using anti-His and anti-OmpA sera. Data are representative of four independent experiments.

**e)** Δ*lptM* cells harboring pEVOL-pBpF and $pLptDME^{His}$ derivative (*) forms encoding LptE with *p*Bpa engineered at the indicated positions were subjected to UV irradiation as indicated and envelope fractionation (Load) followed by $LptE^{His}$ affinity purification (Elution). Samples were analyzed by SDS-PAGE and Western blotting using the indicated antisera. Load: 2.2%; Elution: 100%. * indicates LptE amino acid position encoded by the engineered amber codon, except sample "–" that expresses the wild-type LptE. ** indicates a non-identified protein band. Data are representative of three independent experiments.

**a**

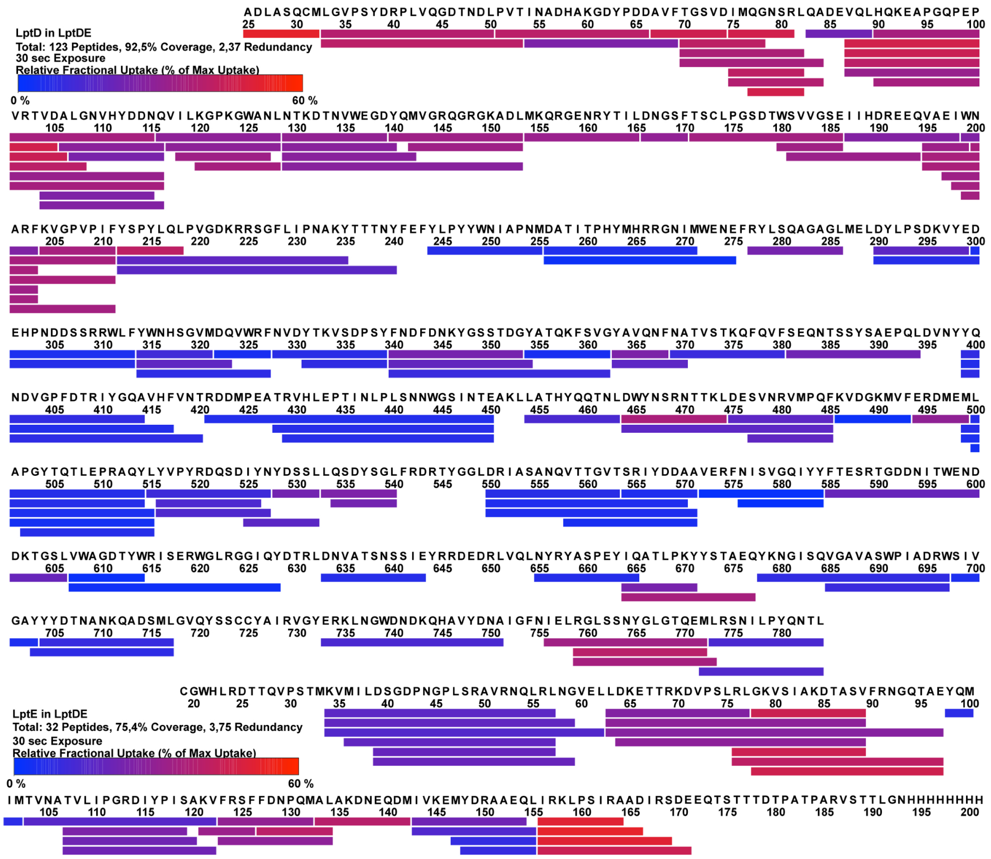

ADLASQCMLGVPSYDRPLVQGDTNDLPVTINADHAKGDYPDDAVFTGSVDIMQGNSRLQADEVQLHQKEAPGQPEP
LptD in LptDE
Total: 123 Peptides, 92,5% Coverage, 2,37 Redundancy
30 sec Exposure
Relative Fractional Uptake (% of Max Uptake)
0 %
60 %
VRTVDALGNVHYDDNQVILKGPKGWANLNTKDTNVWEGDYQMVGRQGRGKADLMKQRGENRYTILDNGSFTSCLPGSDTWSVVGSEIIHDREEQVAEIWN
ARFKVGPVPIFYSPYLQLPVGDKRRSGFLIPNAKYTTTNYFEFYLPYYWNIAPNMDATITPHYMHRRGNIMWENEFRYLSQAGAGLMELDYLPSDKVYED
EHPNDDSSRRWLFYWNHSGVMDQVWRFNVDYTKVSDPSYFNDFDNKYGSSTDGYATQKFSVGYAVQNFNATVSTKQFQVFSEQNTSSYSAEPQLDVNYYQ
NDVGPFDTRIYGQAVHFVNTRDDMPEATRVHLEPTINLPLSNNWGSINTEAKLLATHYQQTNLDWYNSRNTTKLDESVNRVMPQFKVDGKMVFERDMEML
APGYTQTLEPRAQYLYVPYRDQSDIYNYDSSLLQSDYSGLFRDRTYGGLDRIASANQVTTGVTSRIYDDAAVERFNISVGQIYYFTESRTGDDNITWEND
DKTGSLVWAGDTYWRISERWGLRGGIQYDTRLDNVATSNSSIEYRRDEDRLVQLNYRYASPEYIQATLPKYYSTAEQYKNGISQVGAVASWPIADRWSIV
GAYYYDTNANKQADSMLGVQYSSCCYAIRVGYERKLNGWDNDKQHAVYDNAIGFNIELRGLSSNYGLGTQEMLRSNILPYQNTL
CGWHLRDTTQVPSTMKVMILDSGDPNGPLSRAVRNQLRLNGVELLDKETTRKDVPSLRLGKVSIAKDTASVFRNGQTAEYQM
LptE in LptDE
Total: 32 Peptides, 75,4% Coverage, 3,75 Redundancy
30 sec Exposure
Relative Fractional Uptake (% of Max Uptake)
0 %
60 %
IMTVNATVLIPGRDIYPISAKVFRSFFDNPQMALAKDNEQDMIVKEMYDRAAEQLIRKLPSIRAADIRSDEEQTSTTTDTPATPARVSTTLGNHHHHHHHH


**b**

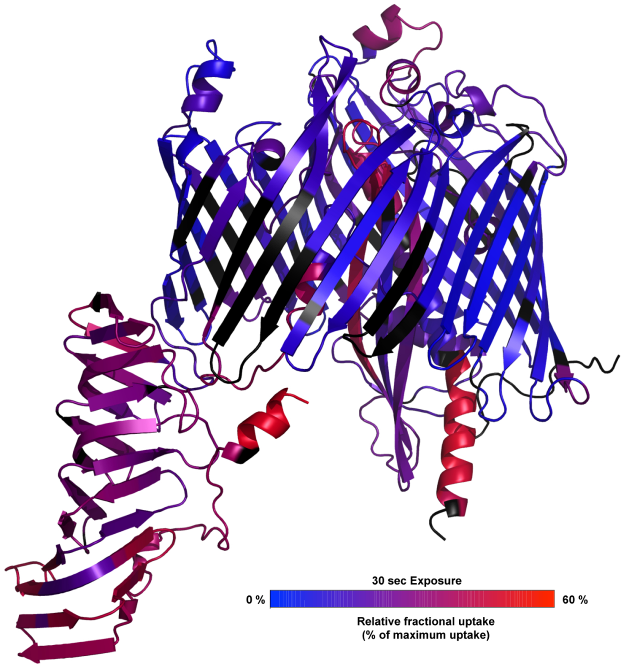

30 sec Exposure
0 %
60 %
Relative fractional uptake
(% of maximum uptake)

**Supplementary Figure 9**. **HDX-MS of the LptDE translocon.**

**a)** Sequence coverage maps of LptD (top) and LptE (bottom) obtained by HDX-MS of LptD and LptE. The color scale shows the relative deuterium uptake after 30 sec of deuteration from 0% (blue) to 60% (red).

**b)** Relative deuterium uptake of LptDE after 30 sec deuteration color coded from 0% (blue) to 60% (red) on the three-dimensional structure of LptDE, as predicted by our AlphaFold2 model.

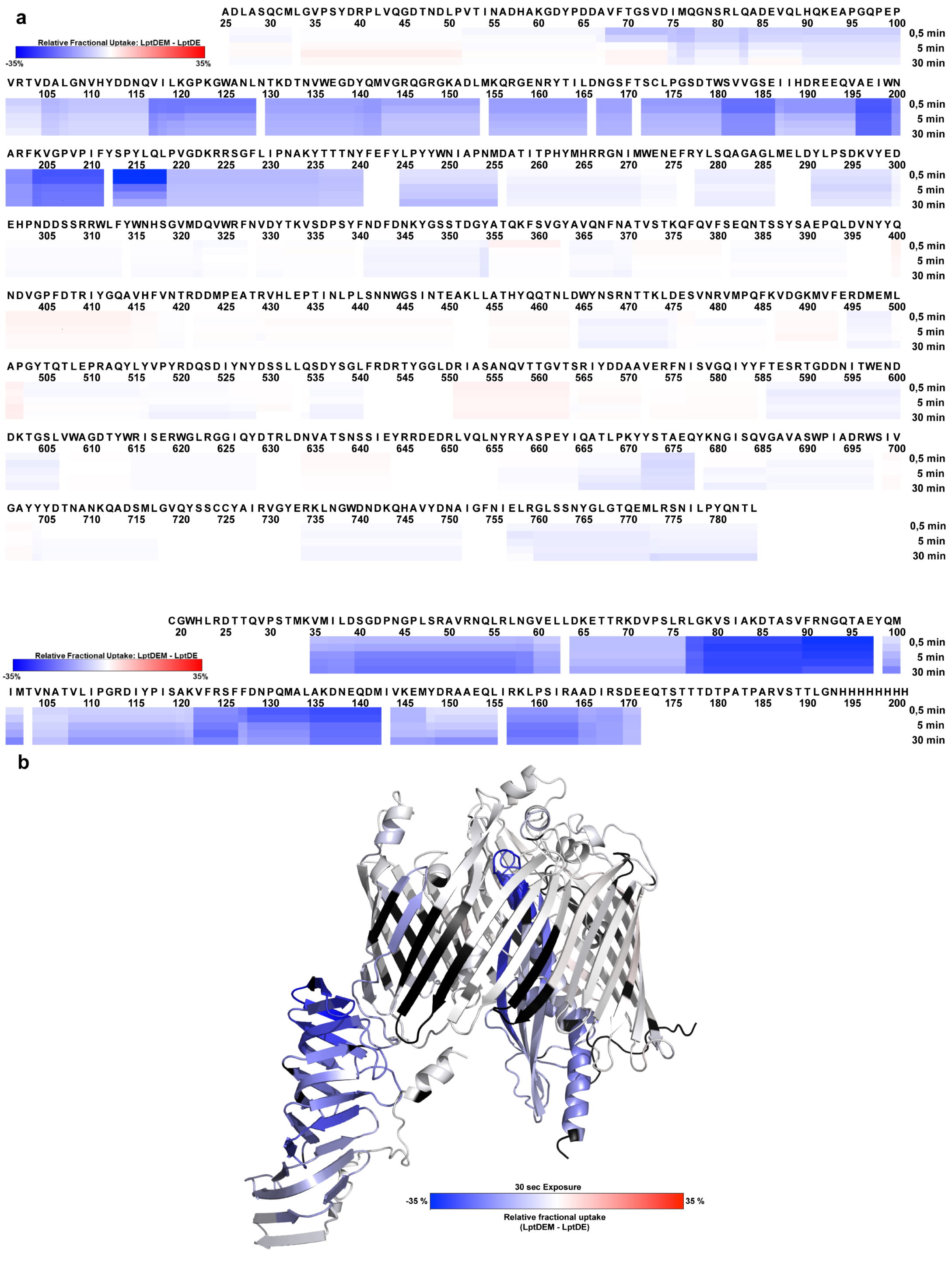

**Supplementary Figure 10. Deuteration heatmap of LptD and LptE**

**a)** Differential heatmap of LptD (top) and LptE (bottom) between the LptDE trans-locon in the presence or absence of LptM color coded, -35% (blue: protected in the complex)/0% (white: no difference)/+35% (red: deprotected in the complex).
**b)** The color coded heatmap described in (a, timepoint 30 sec) is represented on the three-dimensional structure of LptDE, as predicted by our AlphaFold2 model.

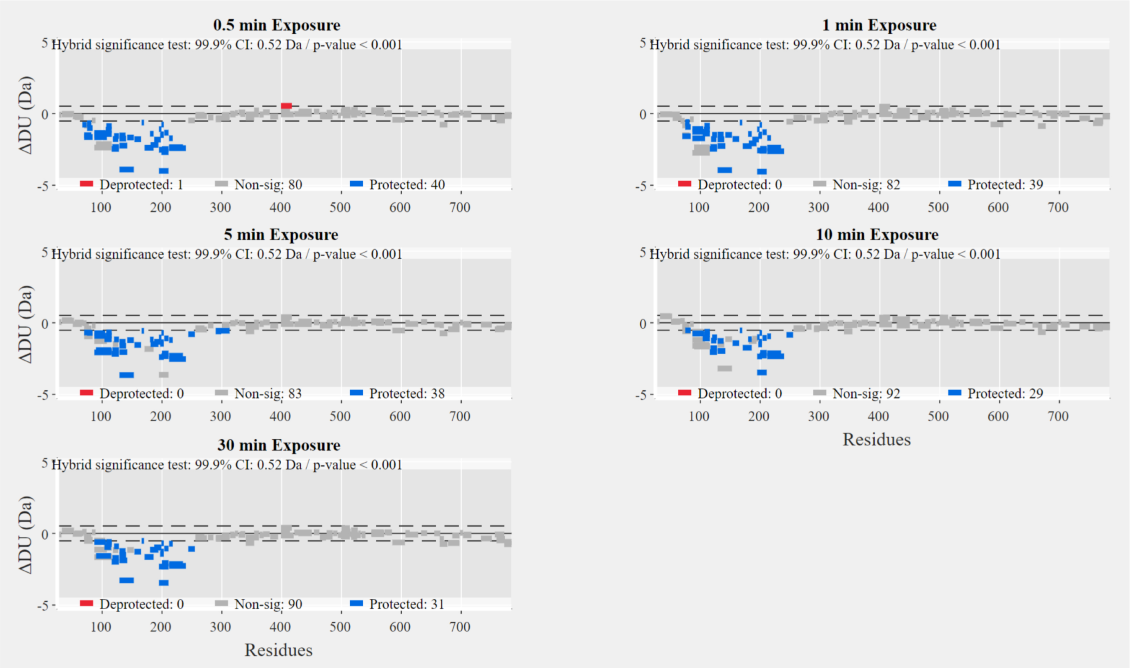
0.5 min Exposure
Hybrid significance test: 99.9% CI: 0.52 Da / p-value < 0.001
Deprotected: 1
Non-sig: 80
Protected: 40
1 min Exposure
Hybrid significance test: 99.9% CI: 0.52 Da / p-value < 0.001
Deprotected: 0
Non-sig: 82
Protected: 39
5 min Exposure
Hybrid significance test: 99.9% CI: 0.52 Da / p-value < 0.001
Deprotected: 0
Non-sig: 83
Protected: 38
10 min Exposure
Hybrid significance test: 99.9% CI: 0.52 Da / p-value < 0.001
Deprotected: 0
Non-sig: 92
Protected: 29
30 min Exposure
Hybrid significance test: 99.9% CI: 0.52 Da / p-value < 0.001
Deprotected: 0
Non-sig: 90
Protected: 31
ΔDU (Da)
Residues

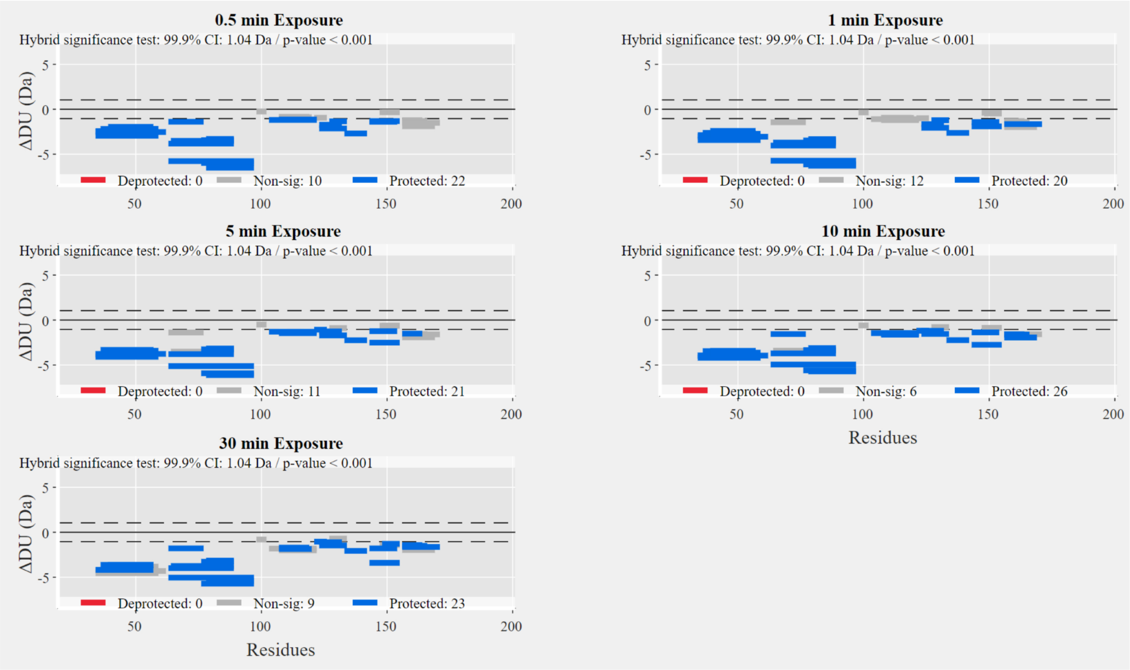
0.5 min Exposure
Hybrid significance test: 99.9% CI: 1.04 Da / p-value < 0.001
Deprotected: 0
Non-sig: 10
Protected: 22
1 min Exposure
Hybrid significance test: 99.9% CI: 1.04 Da / p-value < 0.001
Deprotected: 0
Non-sig: 12
Protected: 20
5 min Exposure
Hybrid significance test: 99.9% CI: 1.04 Da / p-value < 0.001
Deprotected: 0
Non-sig: 11
Protected: 21
10 min Exposure
Hybrid significance test: 99.9% CI: 1.04 Da / p-value < 0.001
Deprotected: 0
Non-sig: 6
Protected: 26
30 min Exposure
Hybrid significance test: 99.9% CI: 1.04 Da / p-value < 0.001
Deprotected: 0
Non-sig: 9
Protected: 23
ΔDU (Da)
Residues

**Supplementary Figure 11. LptDE heterodimer and LptDEM heterotrimer deuteration Wood Plots.**

Differential Woods Plots between LptDE heterodimer and LptDEM heterotrimer, at the different time points of deuteration (0.5, 1, 5, 10 and 30 min), showing in blue and red, peptides that are significantly protected and deprotected, respectively (hybrid significance test, p-value<0.001, Confidence Intervals 0.52 Da and 1.04 for LptD and LptE, respectively).

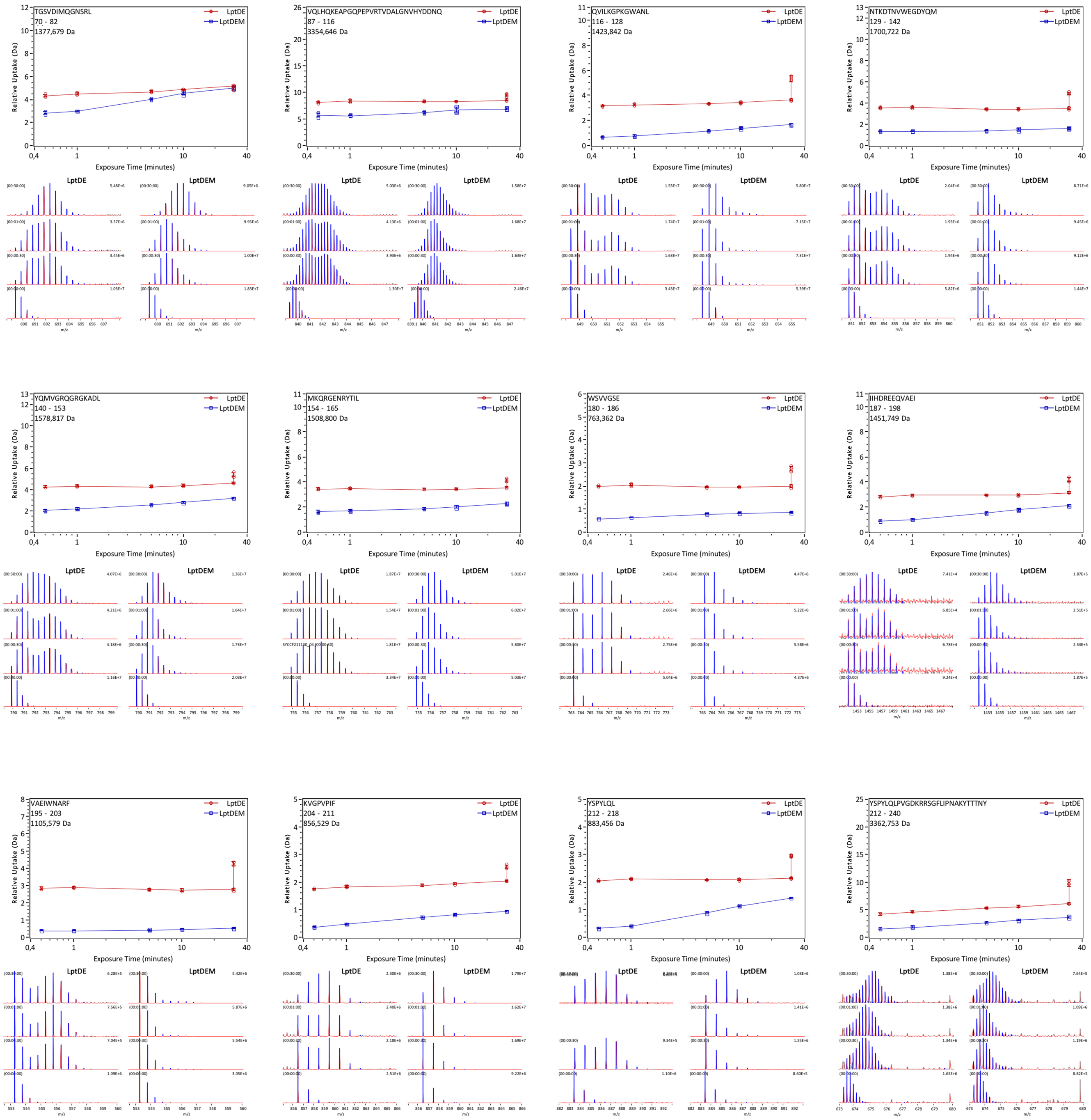

TGSVDIMQGNSRL
70 - 82
1377,679 Da
VQLHQKEAPGQPEPVRTVDALGNVHYDDNQ
87 - 116
3354,646 Da
QVILKGPKGWANL
116 - 128
1423,842 Da
NTKDTNVWEGDYQM
129 - 142
1700,722 Da
YQMVGRQGRGKADL
140 - 153
1578,817 Da
MKQRGENRYTIL
154 - 165
1508,800 Da
WSVVGSE
180 - 186
763,362 Da
IIHDREEQVAEI
187 - 198
1451,749 Da
VAEIWNARF
195 - 203
1105,579 Da
KVGPVPIF
204 - 211
856,529 Da
YSPYLQL
212 - 218
883,456 Da
YSPYLQLPVGDKRRSGFLIPNAKYTTTNY
212 - 240
3362,753 Da
LptDE
LptDEM
Relative Uptake (Da)
Exposure Time (minutes)
m/z

**Supplementary Figure 12. Mass spectra of LptD β-taco peptides showing bimodal distributions.**

For each peptide are shown: top, relative deuterium uptake as a function of deuterium exposure time (red line for the LptDE and blue line for the LptDEM samples), maximally deuterated control is represented in triplicate at t = 30 min (in some cases for, e.g peptide [70-82], the uptake of the control overlays with the 30 min timepoint); bottom: mass spectra obtained with LptDE (left) and LptDEM (right).

a

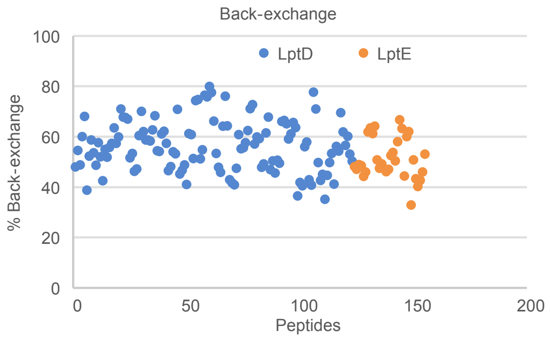


b

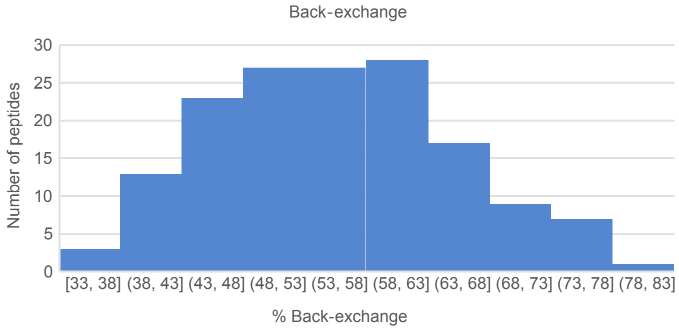


**Supplementary Figure 13. Back-exchange.**

Back-exchange values were calculated for each peptide of LptD and LptE (as indicated by colour code) and represented as a scatter plot **(a)** and bar plot **(b)**. These values range from 35,1% to 79.9%, with an average of 54.9%.

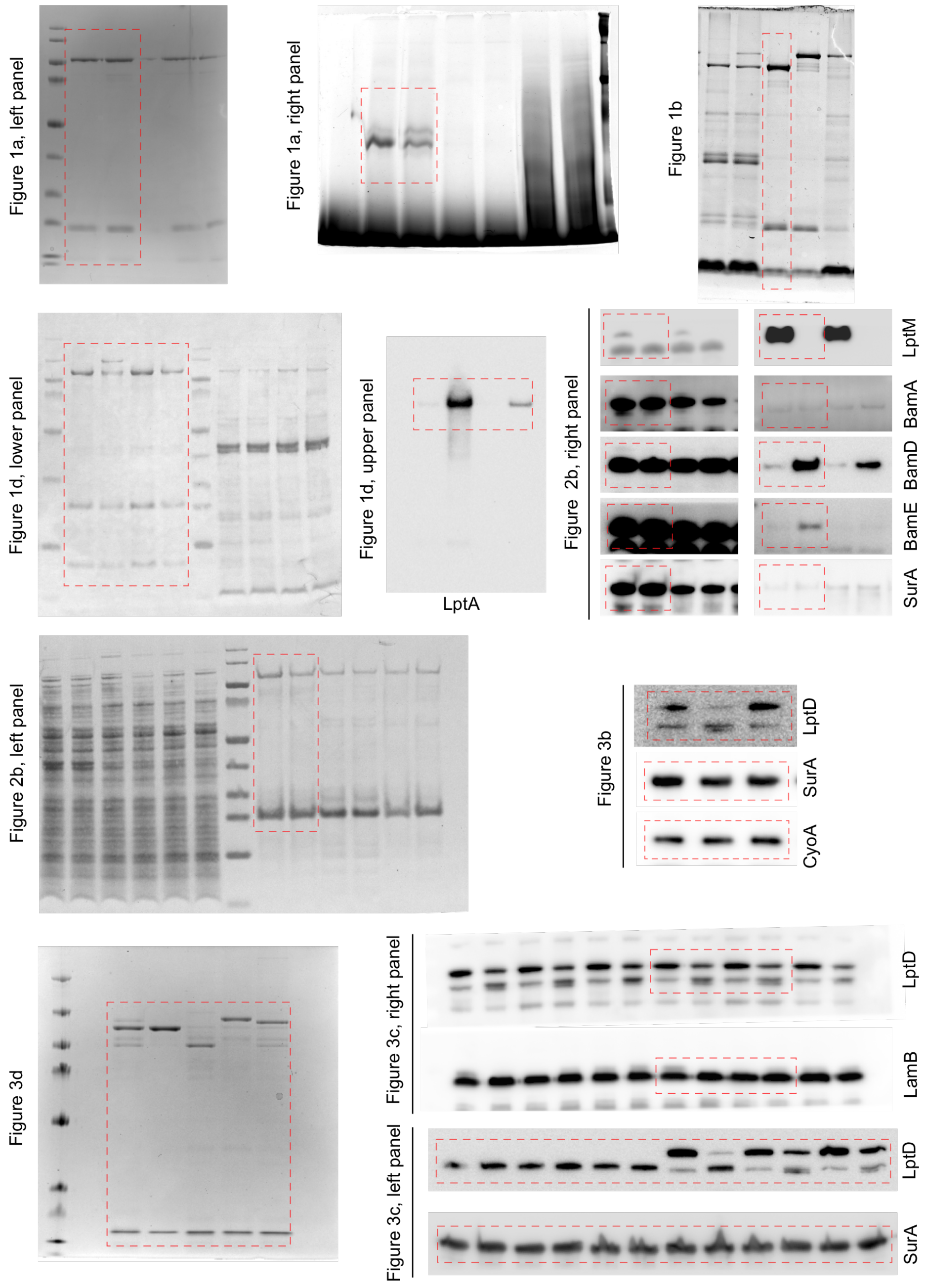


**Supplementary Figure 14. Uncropped gels and membranes.**

Red boxes indicate areas cropped and presented in main figures as indicated.

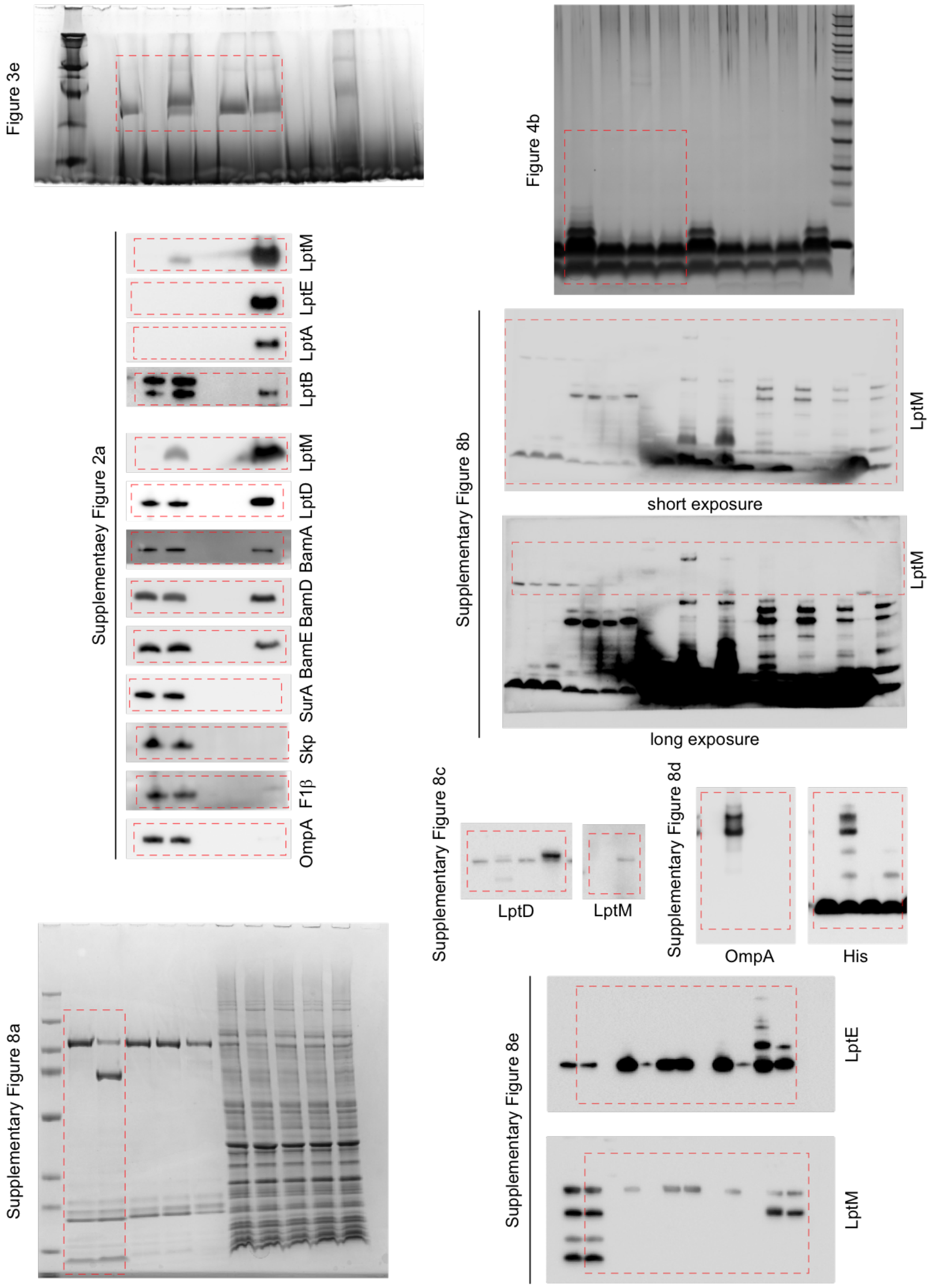


**Supplementary Figure 15. Uncropped gels and membranes.**

Red boxes indicate areas cropped and presented in main and supplementary figures as indicated.

**Supplementary Table 1. Oligonucleotides used in this study**

| Oligonucleotide name | Sequence | Lab Identifier |
|---|---|---|
| pJH-lptM-Fw | GGTTAGGAAGAACGCATAATAAC-GATGAAAAACGTGTTTAAGGCACTCACT | YY37 |
| pJH-lptM-Rev | GTGGTGATGATGGTGGTGATGATGG-TAATTCACCTGGGATGGACCAT | YY38 |
| pJH-lptD-Fw | GGTTAGGAAGAACGCATAATAAC-GATGAAAAAACGTATCCCCACTCTC | YY66 |
| pJH-lptD-lptM-Rev | TGAGTGCCTTAAACAC-GTTTTTCATCCCTCCGCCGGCCGCTGCTCACAAAGTGTTTTGATACGGCAG | YY67 |
| lptM-lptE-Fw | GATGGTCCATCCCAGGTGAATTACTAA-GCAGCGGCCGGCGGAGGGGTGCGA-TATCTGGCAACATT | YY68 |
| lptE-8His-Rev | TTAG-TGGTGATGATGGTGGTGATGATGGTTACCCAGCGTGGTGGAGA | YY69 |
| lptD-lptE-Fw | TCTGCCGTATCAAAACACTTTGTGAG-CAGCGGCCGGCGGAGGGGTGCGA-TATCTGGCAACATTGTTGT | YY81 |
| lptE-lptM-Rev | GTTTTTCATCCCTCCGCCGGCCGCTGCTTAGTTACCCAGCGTGGTGGAGA | YY82 |
| lptD-C31S-Fw | GCCTCACAGTCAATGTTGGGCGTGCC | YY142 |
| lptD-C31S-Rev | GGCACGCCCAACATTGACTGTGAGGCG | YY143 |
| lptD-C173S-Fw | GCTTTACCTCCTCAC-TGCCGGGTTCTGACACC | YY144 |
| lptD-C173S-Rev | GGTGTCAGAACCCGGCAGTGAG-GAGGTAAAGC | YY145 |
| lptD-C724S-Fw | GCAATACAGCTCCTCATGCTATGCAATTCGC | YY146 |
| lptD-C724S-Rev | GCGAATTGCATAGCATGAGGAGCTG-TATTGC | YY147 |
| lptD-C725S-Fw | GCAATACAGCTCCTGCTCATATGCAATTCGC | YY148 |
| lptD-C725S-Rev | GCGAATTGCATATGAGCAGGAGCTG-TATTGC | YY149 |
| lptD-C724/5S-Fw | GCAATACAGCTCCTCATCATATGCAATTCGC | YY150 |
| lptD-C724/5S-Rev | GCGAATTGCATATGATGAGGAGCTGTATTGC | YY151 |
| lptM-L22-Fw1 | CTGACGGGCTGCGGTTA-GAAAGGTCCGCTCTAT | YY178 |
| lptM-L22-Rev1 | ATAGAGCGGACCTTTCTAACCG-CAGCCCGTCAG | YY179 |
| lptM-Y27-Fw2 | CTGACGGGCTGCGGTTA-GAAAGGTCCGCTCTAT | YY180 |
| lptM-Y27-Rev2 | ATAGAGCGGACCTTTCTAACCG-CAGCCCGTCAG | YY181 |
| lptM-V42-Fw4 | CCTGCAGATAAAAACTAGCCGCCGCCGAC- | YY184 |

| | | |
|---|---|---|
| | CAAA | |
| lptM-V42-Rev4 | TTTGGTCGGCGGCGGCTAGTTTTTATCTG-CAGG | YY185 |
| lptM-V50-Fw5 | CCGCCGACCAAACCGTAGGAGACGCAAAC-GCAA | YY186 |
| lptM-V50-Rev5 | TTGCGTTTGCGTCTCCTACGGTTT-GGTCGGCGG | YY187 |
| lptM-A57-Fw6 | CAAACGCAATCCACGTAGCCGGA-TAAAAACGAT | YY188 |
| lptM-A57-Rev6 | ATCGTTTTTATCCGGCTACGTG-GATTGCGTTTG | YY189 |
| lptM-Y67-Fw7 | GATAAAAACGATCGCTA-GACTGGCGATGGTCCA | YY190 |
| lptM-Y67-Rev7 | TGGACCATCGCCAG-TCTAGCGATCGTTTTTATC | YY191 |
| lptE-K70-Fw1 | ATAAAGAAACCACGCGTTAGGAC-GTTCCATCCTTG | Ovio69 |
| lptE-K70-Rev1 | CAAGGATGGAACGTCCTAAC-GCGTGGTTTCTTTAT | Ovio70 |
| lptE-A83-Fw4 | AAGTGAGCATCTAGAAAGATACCGCATCGG | Ovio75 |
| lptE-A83-Rev4 | ATGCGGTATCTTTCTAGATGCTCAC-TTTACCC | Ovio76 |
| lptD-Y63-Fw1 | GAAAGGGGACTAGCCGGATGACGC | Ovio79 |
| lptD-Y63-Rev1 | GCGTCATCCGGCTAGTCCCCTTTCGC | Ovio80 |
| lptD-Y347-Fw2 | CGATAACAAGTAGGGTTCCAGTACTGACGG | Ovio81 |
| lptD-Y347-Rev2 | GTAGCCGTCAGTACTGGAACCCTACTTGTTATCG | Ovio82 |
| pBAD-Rev | CATGAATTCCTCCTTTCACTCCATCC | Ovio37 |
| pBAD-Fw | AGCTTGGCTGTTTTGGCGG | Ovio38 |
| pBAD-DsbC-Fw | AAGGAGGAATTCATGAA-GAAAGGTTTTATGTTGTTTACTTTGTTAGCG | Ovio49 |
| pBAD-DsbC-Rev | CAAAACAGCCAAGCTTTATTTAC-CGCTGGTCATTTTTTGGTGTTCG | Ovio50 |
| ∆Nter-LptD-Rev | TGCCAGTCCCTGTTGACTATAAAGGG | Ovio51 |
| ∆Nter-LptD-Fw | TTTAAGGTGGGTCCGGTACCGATC | Ovio52 |

**Supplementary Table 2. HDX data summary**

| Data | LptDE | LptDEM |
|---|---|---|
| HDX reaction details | 95% $D_2O$, pH 2.3, 10°C | |
| HDX time course (min) | 0, 0.5, 1, 5, 10, 30 | |
| HDX controls | Maximally labelled control in 8M $d_4$-urea in $D_2O$ for 24h at 20°C / Blanks injected between each timepoint | |
| Back-exchange | 54.4 ± 10.0 % | |
| Number of peptides | 121 for LptD and 32 for LptE | |
| Sequence coverage | 92.50% for LptD and 75.41% for LptE | |
| Average peptide Length / Redundancy | 13.52/2.33 for LptD and 16.19/3.75 for LptE | |
| Replicates (technical) | 3 | |
| Repeatability (average SD) | 0.0437 for LptD and 0.0850 for LptE | 0.0492 for LptD and 0.1137 for LptE |
| Significant differences in HDX | hybrid significance test, p-value<0,001, Confidence Interval 0.52 Da for LptD and 1.04 for LptE | |

**Supplementary Table 3. MD data summary**

| Protein model | Non-protein molecules | Atoms | Input box (nm) | Sim. length | Repeats |
|---|---|---|---|---|---|
| LptD, LptE and LptM | 50 LPS-A, 39 POPG, 78 POPE, 13 cardiolipin | 168,549 | 11.0 x 11.0 x 13.5 | 500 ns | 3 |
| LptD and LptE | 50 LPS-A, 39 POPG, 78 POPE, 13 cardiolipin | 167,691 | 10.9 x 10.9 x 13.6 | 500 ns | 3 |

## Supplementary References


1 Parks, D. H., Chuvochina, M., Waite, D. W. *et al.* A standardized bacterial taxonomy based on genome phylogeny substantially revises the tree of life. *Nature biotechnology* **36**, 996-1004, doi:10.1038/nbt.4229 (2018).

2 Seemann, T. Prokka: rapid prokaryotic genome annotation. *Bioinformatics (Oxford, England)* **30**, 2068-2069, doi:10.1093/bioinformatics/btu153 (2014).

3 Eddy, S. R. Accelerated Profile HMM Searches. *PLoS computational biology* **7**, e1002195, doi:10.1371/journal.pcbi.1002195 (2011).

4 Hauser, M., Steinegger, M. & Söding, J. MMseqs software suite for fast and deep clustering and searching of large protein sequence sets. *Bioinformatics (Oxford, England)* **32**, 1323-1330, doi:10.1093/bioinformatics/btw006 (2016).

5 Traag, V. A., Waltman, L. & van Eck, N. J. From Louvain to Leiden: guaranteeing well-connected communities. *Scientific reports* **9**, 5233, doi:10.1038/s41598-019-41695-z (2019).

6 Price, M. N., Dehal, P. S. & Arkin, A. P. FastTree: computing large minimum evolution trees with profiles instead of a distance matrix. *Molecular biology and evolution* **26**, 1641-1650, doi:10.1093/molbev/msp077 (2009).

7 Wagih, O. ggseqlogo: a versatile R package for drawing sequence logos. *Bioinformatics (Oxford, England)* **33**, 3645-3647, doi:10.1093/bioinformatics/btx469 (2017).

8 Bailey, T. L., Boden, M., Buske, F. A. *et al.* MEME SUITE: tools for motif discovery and searching. *Nucleic acids research* **37**, W202-208, doi:10.1093/nar/gkp335 (2009).

9 Adeolu, M., Alnajar, S., Naushad, S. *et al.* Genome-based phylogeny and taxonomy of the 'Enterobacteriales': proposal for Enterobacterales ord. nov. divided into the families Enterobacteriaceae, Erwiniaceae fam. nov., Pectobacteriaceae fam. nov., Yersiniaceae fam. nov., Hafniaceae fam. nov., Morganellaceae fam. nov., and Budviciaceae fam. nov. *International journal of systematic and evolutionary microbiology* **66**, 5575-5599, doi:10.1099/ijsem.0.001485 (2016).

10 Alnajar, S. & Gupta, R. S. Phylogenomics and comparative genomic studies delineate six main clades within the family Enterobacteriaceae and support the reclassification of several polyphyletic members of the family. *Infection, genetics and evolution : journal of molecular epidemiology and evolutionary genetics in infectious diseases* **54**, 108-127, doi:10.1016/j.meegid.2017.06.024 (2017).

11 Pettersen, E. F., Goddard, T. D., Huang, C. C. *et al.* UCSF Chimera--a visualization system for exploratory research and analysis. *Journal of computational chemistry* **25**, 1605-1612, doi:10.1002/jcc.20084 (2004).
