# Supplementary material for: LptM promotes oxidative maturation of the lipopolysaccharide translocon by substrate binding mimicry
Source: Nat Commun. 2023 Oct 11;14:6368. doi: 10.1038/s41467-023-42007-w (PMC10567701; doi:10.1038/s41467-023-42007-w)
Supplement: Supplementary file 2 — Reporting Summary [file 41467_2023_42007_MOESM2_ESM.pdf]

nature portfolio

Corresponding author(s): Raffaele Ieva

Last updated by author(s): Sep 15, 2023

Reporting Summary

Nature Portfolio wishes to improve the reproducibility of the work that we publish. This form provides structure for consistency and transparency in reporting. For further information on Nature Portfolio policies, see our Editorial Policies and the Editorial Policy Checklist.

Statistics

For all statistical analyses, confirm that the following items are present in the figure legend, table legend, main text, or Methods section.

n/a	Confirmed	
☐	☒	The exact sample size (*n*) for each experimental group/condition, given as a discrete number and unit of measurement
☐	☒	A statement on whether measurements were taken from distinct samples or whether the same sample was measured repeatedly
☐	☒	The statistical test(s) used AND whether they are one- or two-sided<br>*Only common tests should be described solely by name; describe more complex techniques in the Methods section.*
☒	☐	A description of all covariates tested
☒	☐	A description of any assumptions or corrections, such as tests of normality and adjustment for multiple comparisons
☐	☒	A full description of the statistical parameters including central tendency (e.g. means) or other basic estimates (e.g. regression coefficient) AND variation (e.g. standard deviation) or associated estimates of uncertainty (e.g. confidence intervals)
☐	☒	For null hypothesis testing, the test statistic (e.g. *F*, *t*, *r*) with confidence intervals, effect sizes, degrees of freedom and *P* value noted<br>*Give P values as exact values whenever suitable.*
☒	☐	For Bayesian analysis, information on the choice of priors and Markov chain Monte Carlo settings
☒	☐	For hierarchical and complex designs, identification of the appropriate level for tests and full reporting of outcomes
☒	☐	Estimates of effect sizes (e.g. Cohen's *d*, Pearson's *r*), indicating how they were calculated

Our web collection on statistics for biologists contains articles on many of the points above.

Software and code

Policy information about availability of computer code

Data collection	Chemiluminescence signals were acquired using a LAS 4000 (Fujifilm) imager and software.<br>MALDI-TOF/TOF mass-spectrometry were acquired using a Data Explorer software (Applied Biosystems).<br>Native and HDX MS data were acquired on a Synapt-G2Si with MassLynx 4.1 (Triversa Nanomate, Advion Biosciences).<br>Peptide identification was performed with ProteinLynx Global SERVER (PLGS, Waters Scientific).<br>Protein structure prediction data were acquired using AlphaFold Multimer v1.0.<br>The models were built into simulation systems using CHARMM-GUI.
Data analysis	The following software are referenced in our manuscript:<br>Multi Gauge software (Fujifilm)<br>Deuteros 2.0<br>PyMOL (The PyMOL Molecular Graphics System, Version 2.3.0 Schrödinger)<br>DynamX 3.0<br>HDX-Viewer<br>PropKa3.1<br>Gromacs 2020.1<br>VMD 1.9.4a43<br>Matplotlib<br>Prokka<br>HMMER

Fasttree
Ggseqlogo
Mmseqs2
Igraph package
Meme
Mast
IQ-TREE

For manuscripts utilizing custom algorithms or software that are central to the research but not yet described in published literature, software must be made available to editors and reviewers. We strongly encourage code deposition in a community repository (e.g. GitHub). See the Nature Portfolio guidelines for submitting code & software for further information.

Data

Policy information about availability of data

All manuscripts must include a data availability statement. This statement should provide the following information, where applicable:

- Accession codes, unique identifiers, or web links for publicly available datasets
- A description of any restrictions on data availability
- For clinical datasets or third party data, please ensure that the statement adheres to our policy

Sources data are provided within this paper. The genomes used in this studies, their accession codes and corresponding hyperlinks are listed in the Source Data file. MALDI-TOF/TOF data and quantifications of coomassie-stained gel protein bands in Fig. 3d are available in the Source Data file. Un-cropped gels are available in Supplementary Figs. 14 and 15 of the Supplementary Information file. Native- and HDX-MS data are available via the PRIDE partner repository under the accession code PXD041774 [http://proteomecentral.proteomexchange.org/cgi/GetDataset?ID=PXD041774]. Structural models are available via the Open Science Framework under the accession code xpfjc [https://osf.io/xpfjc/].

Research involving human participants, their data, or biological material

Policy information about studies with human participants or human data. See also policy information about sex, gender (identity/presentation), and sexual orientation and race, ethnicity and racism.

Reporting on sex and gender	not relevant
Reporting on race, ethnicity, or other socially relevant groupings	not relevant
Population characteristics	not relevant
Recruitment	not relevant
Ethics oversight	not relevant

Note that full information on the approval of the study protocol must also be provided in the manuscript.

Field-specific reporting

Please select the one below that is the best fit for your research. If you are not sure, read the appropriate sections before making your selection.

[x] Life sciences [ ] Behavioural & social sciences [ ] Ecological, evolutionary & environmental sciences

For a reference copy of the document with all sections, see nature.com/documents/nr-reporting-summary-flat.pdf

Life sciences study design

All studies must disclose on these points even when the disclosure is negative.

Sample size	No sample size determination was needed. The number of independent experiments was determined based on the level of variability within replicates and based on the experience with each methodology. Independent triplicates or quadruplicates (indicated in Figure Legends and Methods) were performed for each experiment as standards for the techniques used.
Data exclusions	No data exclusion was needed.
Replication	Each experiment was repeated at least three times. All attempts at replication were successful. In the case of protein gels, representative results are shown.
Randomization	Randomization is not relevant to our analyses as there were no covariates applicable within this study.
Blinding	Blinding was not required, as it is not necessary for the techniques used.

Reporting for specific materials, systems and methods

We require information from authors about some types of materials, experimental systems and methods used in many studies. Here, indicate whether each material, system or method listed is relevant to your study. If you are not sure if a list item applies to your research, read the appropriate section before selecting a response.

Materials & experimental systems

n/a	Involved in the study	
☐	☒	Antibodies
☒	☐	Eukaryotic cell lines
☒	☐	Palaeontology and archaeology
☒	☐	Animals and other organisms
☒	☐	Clinical data
☒	☐	Dual use research of concern
☒	☐	Plants

Methods

n/a	Involved in the study	
☒	☐	ChIP-seq
☒	☐	Flow cytometry
☒	☐	MRI-based neuroimaging

Antibodies

Antibodies used	Antisera were raised in rabbits against peptides or full proteins from Escherichia coli (LptM, dilution 1:1000; BamA, 1:1000; BamD, 1:1000; BamE, 1:1000; SurA, dilution 1:1000; Skp, 1:1000; OmpA, 1:1000; CyoA, 1:1000; LptD, 1:5000; LptE, 1:10000; LptA, 1:1000; LptB, 1:10000) or Saccharomyces cerevisiae (F1beta, 1:1000). Horseradish peroxidase conjugated anti-polyhistidine (TaKaRa product n. 631210, 1:2000) and horseradish peroxidase conjugated anti-rabbit IgG (Sigma product n. A6154, 1:10000) were purchased.
Validation	Each antibody was tested for specificity by SDS-PAGE and western blotting, comparing signals obtained from cell lysates of E. coli deletion stains or E. coli strains expressing truncated or tagged versions of the proteins of interest. Absence or shift of the protein band signals (in lysates obtained from mutant strains with respect to a lysate obtained from a wild-type reference strain) proved the specificity of antisera.
