# Supplementary material for: LptM promotes oxidative maturation of the lipopolysaccharide translocon by substrate binding mimicry
Source: Nat Commun. 2023 Oct 11;14:6368. doi: 10.1038/s41467-023-42007-w (PMC10567701; doi:10.1038/s41467-023-42007-w)
Supplement: Supplementary file 3 — Description of Additional Supplementary Files [file 41467_2023_42007_MOESM3_ESM.pdf]

Title: Supplementary Data 1.
Description: Protein C-alpha RMSD. Protein C-alpha root mean square deviations (RMSDs) for the LptDE and LptDEM complexes over 3 x 500 ns simulations. In all cases the RMSDs converge to ca. 0.3 nm, suggesting that the input models are stable.

Title: Supplementary Data 2.
Description: LptD Deuteration kinetics.

Relative deuterium uptake plot as a function of deuterium exposure time for each peptide of LptD in LptDE (red line) and LptDEM (blue line). Maximally deuterated control is represented in triplicate at t = 30 min (in some cases for, *e.g.* peptide [494-499], the uptake of the control overlays with the 30 min timepoint).

Title: Supplementary Data 3.
Description: LptE Deuteration kinetics.

Relative deuterium uptake plot as a function of deuterium exposure time for each peptide of LptE in LptDE (red line) and LptDEM (blue line). Maximally deuterated control is represented in triplicate at t = 30 min (in some cases for, *e.g.* peptide [123-132], the uptake of the control overlays with the 30 min time point).

Title: Supplementary Data 4.
Description: List of protein sequences similar to LptM in proteobacteria

Title: Supplementary Data 5.

Description: List of protein sequences similar to LptM in non-proteobacteria

Title: Supplementary Data 6.

Description: List of protein sequences similar to LptM in *Enterobacteriaceae*
