# Supplementary figures and images for: LptM promotes oxidative maturation of the lipopolysaccharide translocon by substrate binding mimicry
Source: Nat Commun. 2023 Oct 11;14:6368. doi: 10.1038/s41467-023-42007-w (PMC10567701; doi:10.1038/s41467-023-42007-w)

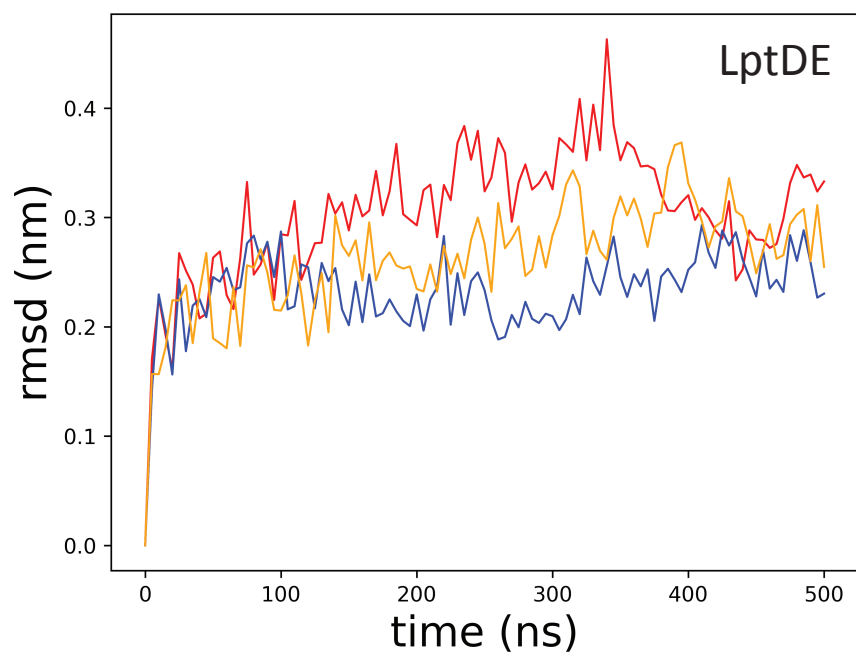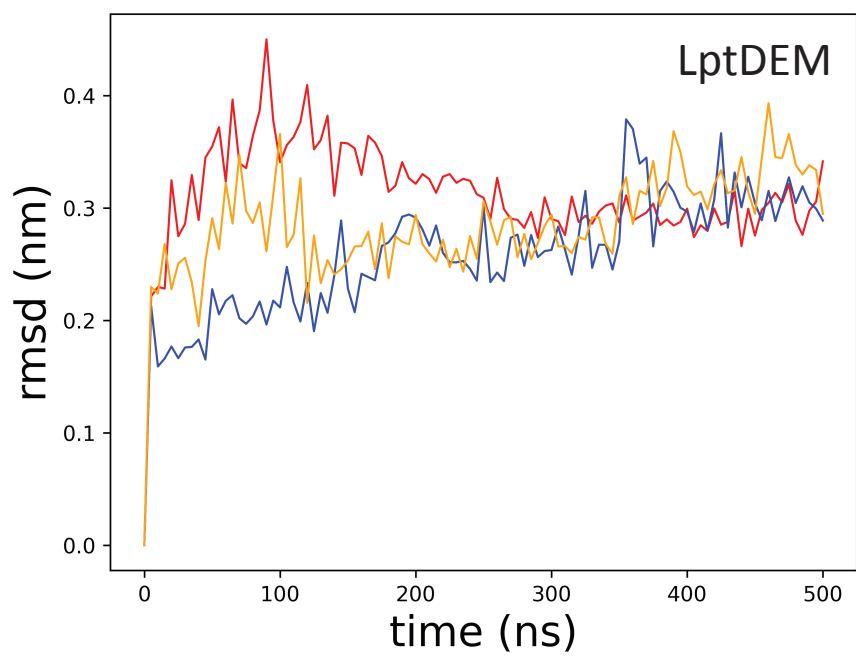

Supplement: Supplementary file 4 — Supplementary Dataset 1 [file 41467_2023_42007_MOESM4_ESM.pdf]

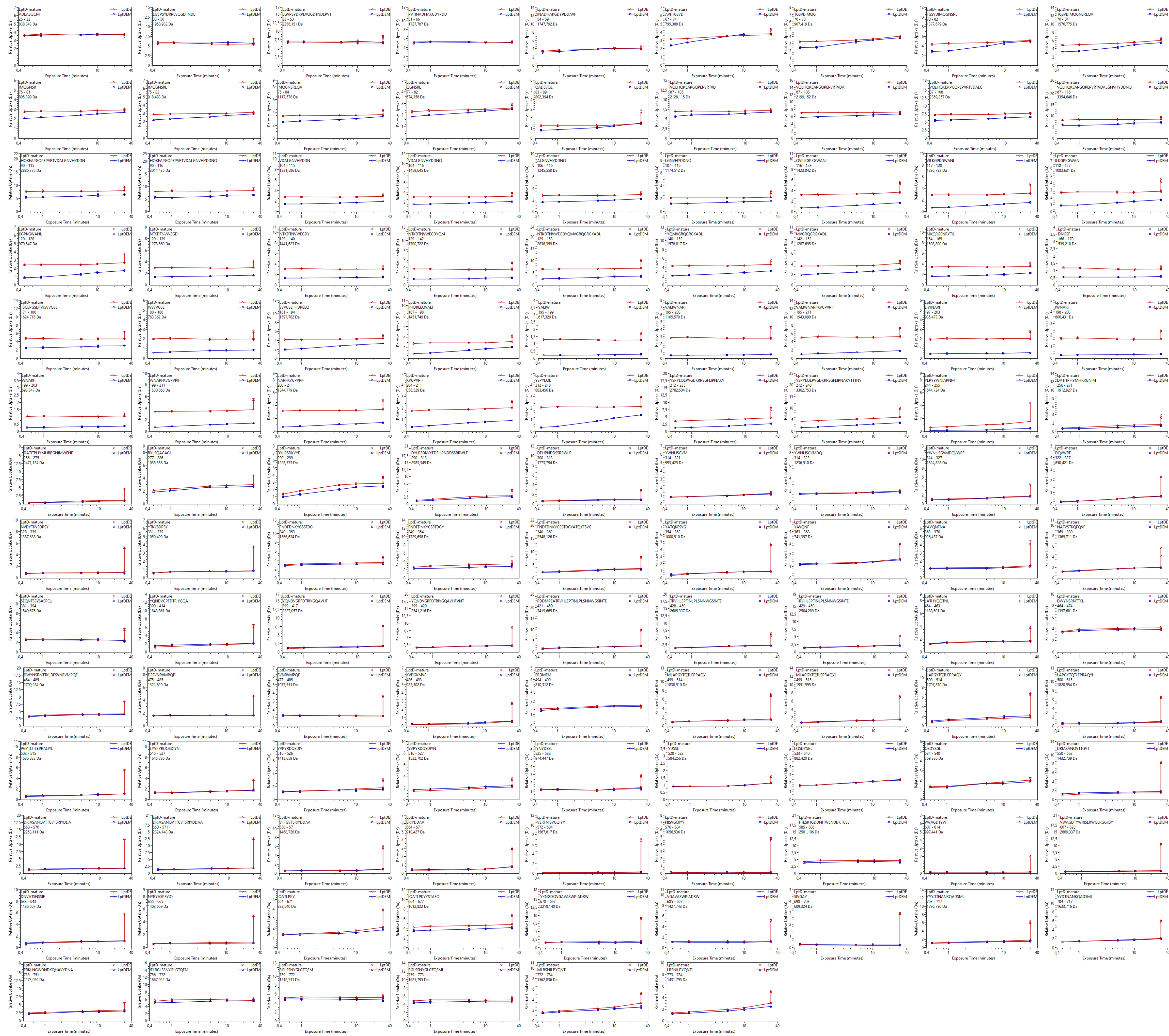

Supplement: Supplementary file 5 — Supplementary Dataset 2 [file 41467_2023_42007_MOESM5_ESM.pdf]

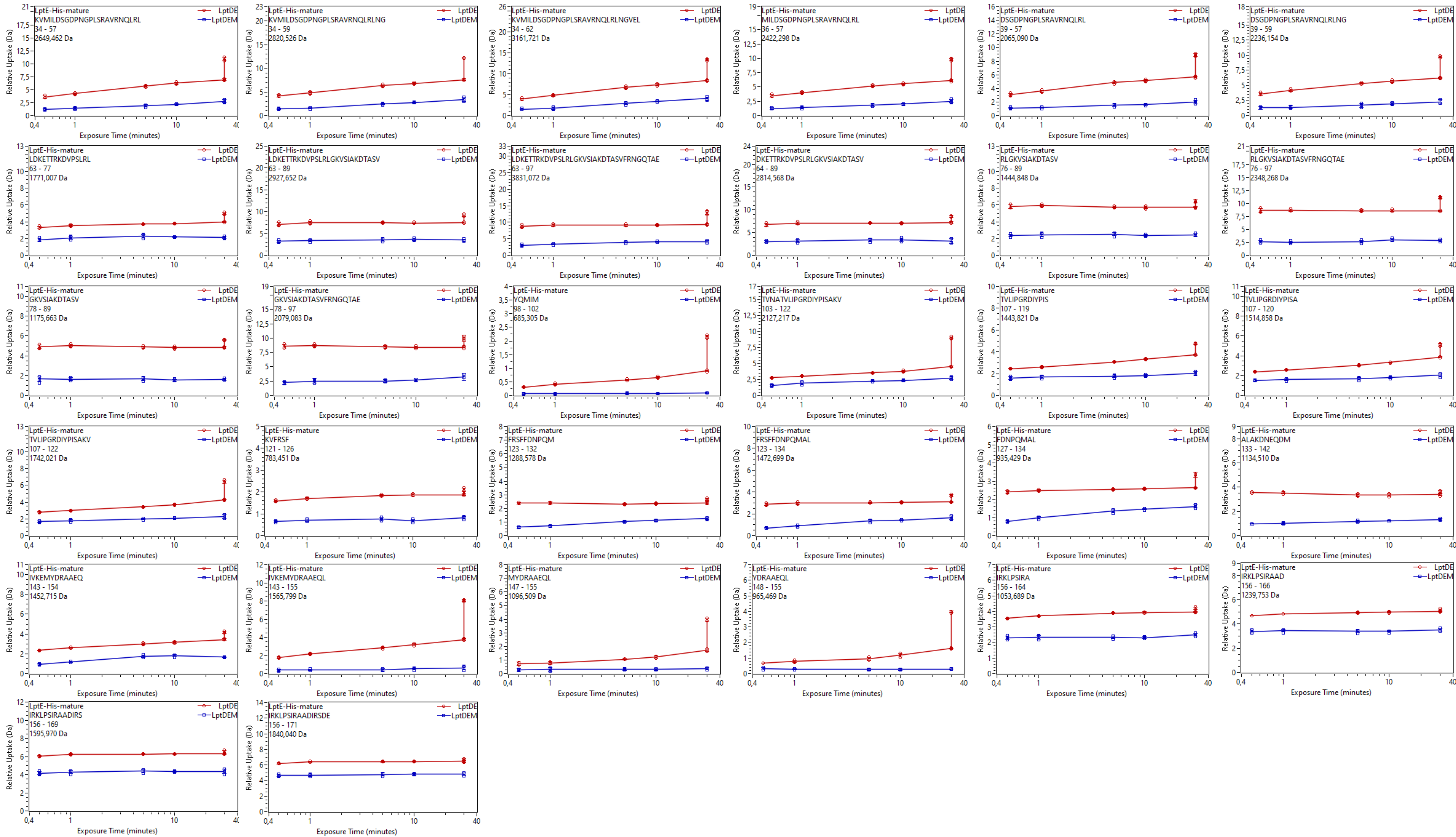

Supplement: Supplementary file 6 — Supplementary Dataset 3 [file 41467_2023_42007_MOESM6_ESM.pdf]
